# Safety and Immunogenicity of the Heterosubtypic Influenza A Vaccine MVA-NP+M1 Manufactured on the AGE1.CR.pIX Avian Cell Line

**DOI:** 10.3390/vaccines7010033

**Published:** 2019-03-22

**Authors:** Pedro M. Folegatti, Duncan Bellamy, Amy Flaxman, Catherine Mair, Chris Ellis, Raquel L. Ramon, Fernando Ramos Lopez, Celia Mitton, Megan Baker, Ian Poulton, Alison Lawrie, Rachel Roberts, Angela Minassian, Katie J. Ewer, Thomas G. Evans, Adrian V. S. Hill, Sarah C. Gilbert

**Affiliations:** 1 The Jenner Institute, University of Oxford, ORCRB, Roosevelt Drive, Oxford OX3 7DQ, UK; duncan.bellamy@ndm.ox.ac.uk (D.B.); amy.flaxman@ndm.ox.ac.uk (A.F.); 2177110m@student.gla.ac.uk (C.M.); raquel.lopezramon@ndm.ox.ac.uk (R.L.R.); fernando.ramoslopez@ndm.ox.ac.uk (F.R.L.); celia.mitton@ndm.ox.ac.uk (C.M.); megan.baker@ndm.ox.ac.uk (M.B.); ian.poulton@ndm.ox.ac.uk (I.P.); alison.lawrie@ndm.ox.ac.uk (A.L.); rachel.roberts@ndm.ox.ac.uk (R.R.); angela.minassian@ndm.ox.ac.uk (A.M.); katie.ewer@ndm.ox.ac.uk (K.J.E.); adrian.hill@ndm.ox.ac.uk (A.V.S.H.); sarah.gilbert@ndm.ox.ac.uk (S.C.G.); 2 Vaccitech Ltd., The Schrodinger Building, 2nd Floor, Oxford Science Park, Heatley Road, Oxford OX4 4GE, UK; chris.ellis@vaccitech.co.uk (C.E.); tom.evans@vaccitech.co.uk (T.G.E.)

**Keywords:** influenza, heterosubtypic vaccine, safety, immunogenicity, viral vectors

## Abstract

Seasonal influenza infections have a significant global impact leading to increased health and economic burden. The efficacy of currently available seasonal influenza vaccines targeting polymorphic surface antigens has historically been suboptimal. Cellular immune responses against highly conserved Influenza A virus antigens, such as nucleoprotein (NP) and matrix protein-1 (M1), have previously been shown to be associated with protection from disease, whilst viral-vectored vaccines are an effective strategy to boost cell-mediated immunity. We have previously demonstrated that MVA encoding NP and M1 can induce potent and persistent T cell responses against influenza. In this Phase I study, we evaluated the safety and immunogenicity of MVA-NP+M1, which was newly manufactured on an immortalized cell line, in six healthy adult participants. The vaccine was well-tolerated with only mild to moderate adverse events that resolved spontaneously and were comparable to previous studies with the same vaccine manufactured in chick embryo fibroblasts. A significant increase in vaccine-specific T cell responses was detected seven days after immunization and was directed against both antigens in the vector insert. This small Phase I study supports progression of this vaccine to a Phase IIb study to assess immunogenicity and additional protective efficacy in older adults receiving licensed seasonal influenza vaccines.

## 1. Introduction

Seasonal influenza has a significant global impact, accounting for an excess mortality rate of up to 6.4 per 100,000 individuals annually in the general population, and estimates suggest significantly higher mortality amongst older age groups [1]. The economic cost to the healthcare system and society is estimated to be, on average, $11.2 billion per year in the United States alone [2], and the unusual increase in influenza cases observed during the 2017/2018 season in the Northern hemisphere illustrates how easily healthcare systems can become overwhelmed during peak illness periods, with further potential negative impacts in the event of a pandemic.

Currently available seasonal influenza vaccines induce antibody responses targeted to the external glycoproteins of the influenza virus. The humoral responses induced by licensed vaccines are subtype-specific and offer limited heterosubtypic protection against novel subtype reassortants or emerging viruses like H5N1 or H7N9 avian influenza. As the circulating virus strains change, the composition of influenza vaccines is assessed, and if necessary, updated annually for both the Northern and Southern hemispheres to match new strains, which arise from antigenic drift on the surface proteins of these seasonal viruses. This need for constant redesign and remanufacture increases the vaccine’s cost, places limitations on supply and critically delays vaccine production [3]. Mismatches between vaccine strain and circulating viruses can lead to highly variable vaccine effectiveness against H1N1 strains. Efficacy against H3N2 remains unacceptably low even for antigenically well-matched vaccines, which may be at least partially attributed to the egg-based vaccine production process [4,5].

Where individuals exposed to a newly arisen influenza virus strain lack protective neutralising antibodies, cross-reactive T-cells against conserved internal antigens of influenza, such as Nucleoprotein (NP) and Matrix 1 (M1), could overcome the limitations of currently available vaccines. This is particularly important in older age groups in whom vaccine efficacy is lower, increasing their risk of severe illness [6,7,8]. Viral vectors are well-known for their ability to elicit cellular immune responses, so we have developed an attenuated orthopoxvirus modified Vaccinia virus Ankara (MVA), which expresses the highly conserved influenza A antigens NP and M1. We have previously demonstrated the safety and immunogenicity of the candidate influenza vaccine MVA-NP+M1 manufactured on chicken embryo fibroblasts (CEF) [9,10,11,12,13]. 

MVA viruses are strongly host-restricted in that most mammalian cells are not fully permissive; therefore, production of MVA vaccines requires CEF cultures prepared from embryonated eggs. Due to their replication-deficient nature, MVA vectors are required at high titres (typically around 10^8^ plaque-forming units [pfu] per dose), which means that efficient production processes are highly desirable. There are several disadvantages of using CEF. As embryonated eggs must be supplied fresh, the continuous introduction of viable material is considered a significant potential source of contamination. The timing of breeding, isolation and processing of embryos may introduce variations in virus yield, limiting the scalability of the process [14]. Chickens also carry a large number of endogenous retroviruses, which could become activated during the production process, and there are concerns about possible shortages of material in case of an avian influenza pandemic [15,16]. In order to overcome these problems, new cell lines were designed and optimised to address the scalability limitations imposed by the CEF manufacturing process [14,17].

The AGE1.CR.pIX cell line was created by immortalisation of Muscovy duck cells through transfection of the E1 genes from the human adenovirus type 5 into primary cells derived from the retina of a duck embryo [17]. Ducks, as opposed to chickens, carry significantly fewer endogenous retroviral inserts. The AGE1.CR.pIX cell line proliferates with an indefinite life span in suspension in serum-free media with zero or low protein content and is highly permissive for MVA, surpassing yields obtained with primary chicken fibroblasts [14,17]. Therefore, the AGE1.CR.pIX cell line is an attractive alternative to CEF for the production of MVA-NP+M1 vaccines.

Here, we present safety and immunogenicity data on the first use of the candidate influenza vaccine MVA-NP+M1 manufactured on the AGE1.CR.pIX duck cell line. These data support clinical development of this candidate through progression to a phase II trial.

## 2. Materials and Methods 

### 2.1. MVA-NP+M1 (AGE1.CR.pIX) Vaccine

The vaccine construct has been described previously and consists of a replication deficient MVA viral vector expressing the NP and M1 antigens from the influenza A virus (H3N2, A/Panama/2007/99) as a single fusion protein [13]. The new batch of vaccine using the AGE1.CR.pIX Muscovy Duck cell line was manufactured by Emergent BioSolutions (Baltimore, MD, USA). An aliquot of AGE1.CR.pIX cells from the Working Cell Bank was expanded through successive passages to provide a 200 L bioreactor suspension culture, and then infected with the MVA-NP+M1 master virus seed stock. After virus release through sonication, the virus was purified by ion-exchange chromatography and Benzonase treatment before concentration and final formulation.

### 2.2. Study Design and Participants

**Figure 1:**
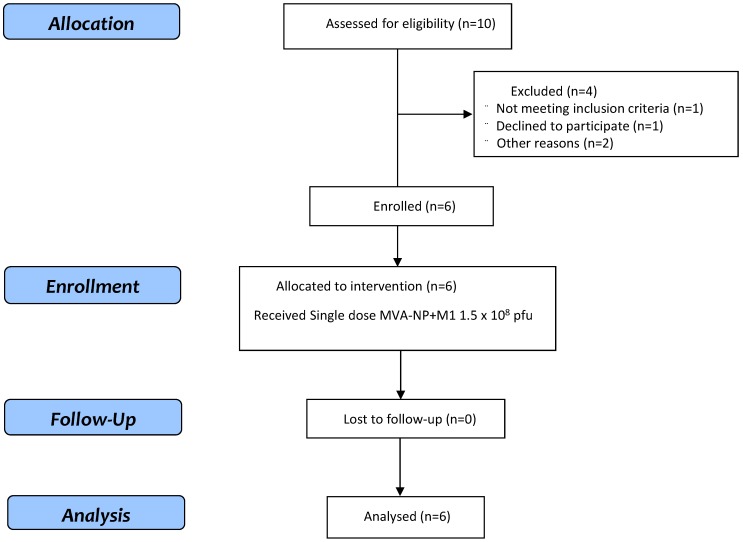
CONSORT flow diagram of the trial.

This is a first-in-human, open-label, non-randomised clinical study of six healthy subjects aged 18–50 years old. Eligible volunteers were recruited at the Centre for Clinical Vaccinology and Tropical Medicine, Oxford, United Kingdom (CONSORT diagram: Figure 1). All participants were healthy adults with negative pre-vaccination tests for HIV antibodies, hepatitis B surface antigen and hepatitis C antibodies. A negative urinary pregnancy test was required at screening and immediately before enrolment for all female subjects. Full details of the eligibility criteria are described in the trial protocol provided in the Supplementary Materials. Written informed consent was obtained in all cases, and the trial was conducted in accordance with the principles of the Declaration of Helsinki and Good Clinical Practice. This study was approved within the UK by the Medicines and Healthcare Products Regulatory Agency (reference 48592/001/001-0001) and the South Centre Berkshire Research Ethics Committee (reference 17/SC/0288). The trial is registered at www.clinicaltrials.gov (Identifier: NCT03277456).

### 2.3. Study Procedures

MVA-NP+M1 (AGE1.CR.pIX) was administered as a single intramuscular injection into the deltoid at a dose of 1.5 × 10^8^ pfu (equivalent to 4.3 × 10^8^ 50% Tissue Culture Infective Dose (TCID50)) in 0.43 mL. A staggered-enrolment approach was used, and interim safety reviews were conducted after vaccination of the first participant; the profile of adverse events (AEs) was then reviewed for the first three volunteers enrolled prior to vaccinations of the remaining subjects in the group. Blood samples were drawn and clinical assessments conducted for safety and/or immunology purposes prior to vaccination at day 0 and subsequently at 2, 7, 21 and 28 days following enrolment. Volunteers were observed in the clinic for one hour after the vaccination procedure and were asked to record any AEs using electronic diaries during the 28-day follow-up period. Swelling at the injection site was objectively assessed by a member of the study team during the study visits. Solicited local site reactions (injection site pain, warmth, redness and pruritus) and systemic symptoms (malaise, myalgia, arthralgia, fatigue, nausea, headache, feverishness and temperature) were recorded for seven days. Unsolicited AEs and serious adverse events (SAEs) were recorded for 28 days. The severity of AEs was graded using the following criteria: (a) mild (short-lived or mild symptoms with no limitation to usual activity); (b) moderate (mild to moderate limitation in usual activity); and (c) severe (considerable limitation in activity, medication or medical attention required).

### 2.4. Endpoints

The primary objective of this trial was to assess the safety and reactogenicity of the candidate influenza vaccine MVA-NP+M1 manufactured on the AGE1.CR.pIX avian cell line. These were assessed based on the occurrence of local and systemic reactogenicity signs and symptoms, the change from baseline for safety laboratory measures and the occurrence of SAEs. The secondary cellular immunogenicity endpoint was assessed by enumerating interferon-gamma (IFN-γ)-producing T cells in an ELISpot assay.

### 2.5. Ex-Vivo IFN-γ ELISpot

Ex vivo IFN-γ enzyme-linked immunosorbent spot (ELISpot) assays were performed using fresh peripheral blood mononuclear cells (PBMCs) to determine responses to the NP+M1 vaccine antigen at days 0 (before vaccination), 7 and 21. Methodology was as described previously [11]. Assays were performed using Multiscreen IP ELISpot plates (Merck Millipore, Watford, UK) coated with 10 μg/mL human anti-IFN-γ antibody and developed using SA-ALP antibody conjugate kits (Mabtech, Stockholm, Sweden) and BCIP NBT-plus chromogenic substrate (Moss Inc., Pasadena, MA, USA). PBMC were separated from whole blood with lithium heparin by density centrifugation within six hours of venepuncture. Cells were incubated for 18–20 hours in RPMI (Sigma) containing 1000 units/mL penicillin, 1 mg/mL streptomycin and 10% heat-inactivated, sterile-filtered foetal calf serum, previously screened for low reactivity (Labtech International, East Sussex, UK). Antigens were tested in triplicate, with 2.5 × 10^5^ PBMC added to each well of the ELISpot plate in a final volume of 100 μL. Results are expressed as spot forming cells (SFC) per million PBMCs, calculated by subtracting the mean negative control response from the mean of each peptide pool response and then summing the response for the eight peptide pools. Each pool contained ten 15–20 mer peptides overlapping by 10 amino acids, spanning the complete NP+M1 vaccine insert. The final concentration of each peptide in the ELISPOT well was 10 μg/mL. Staphylococcal enterotoxin B (0.02 μg/mL) and phytohaemagglutinin-L (10 μg/ mL) were pooled and used as a positive control. Plates were counted using an AID automated ELISpot counter (AID Diagnostika GmbH, algorithm C, Strassberg, Germany) using identical settings for all plates, and counts were adjusted only to remove artefacts. A quality control process was applied where plates were excluded if responses were >80 SFU/million PBMC in the negative control (PBMC without antigen) or <800 SFU/million PBMC in the positive control wells. No plates were excluded for failing QC. Responses to the negative control were low, with a median of 15 SFC (interquartile range (IQR) 8.5–21.75). The lower limit of detection for the assay was 32 SFC for summed responses to NP and M1 pools.

A comparison between T-cell responses in volunteers vaccinated with the MVA-NP+M1 manufactured on the AGE1.CR.pIX cell line versus the vaccine previously manufactured on CEF was conducted using area-under-curve analysis from baseline to week three. Data was used from 21 healthy adult volunteers aged 18–50 years who had been previously vaccinated with 1.5 × 10^8^ pfu MVA-NP+M1 manufactured on CEF [10].

### 2.6. Data Presentation and Statistical Analysis

Safety data is presented according to the frequency, severity and duration of solicited local and systemic reactogenicity signs and symptoms for seven days following vaccination. Unsolicited AEs and SAEs were recorded for four weeks post vaccination. Statistical analysis of immunogenicity data was conducted using GraphPad Prism version 7.03 for Windows (GraphPad Software Inc., San Diego, CA, USA). The area under the curve (AUC) of the T-cell response (IFN-γ SFC/million PBMCs) from baseline to week three, including week one, was calculated using the trapezoidal rule. The median and interquartile range are given for each dataset. Comparisons between datasets were performed using non-parametric tests, as detailed in the figure legend.

## 3. Results

### 3.1. Study Population

A total of six healthy adult volunteers aged 18–50 years (Table 1) were enrolled and administered a single dose of MVA-NP+M1 (AGE1.CR.pIX) intramuscularly at 1.5 × 10^8^ pfu. Volunteers were followed for 28 days post vaccination to assess safety and immunogenicity. Staggered enrolment was applied for the first three volunteers within the group. The demographics of participants within the study are shown in Table 1. The median age was 32.5 years (IQR 24.25–48.5), and two of the six volunteers were male (33.33%).

### 3.2. Vaccine Safety

**Figure 2:**
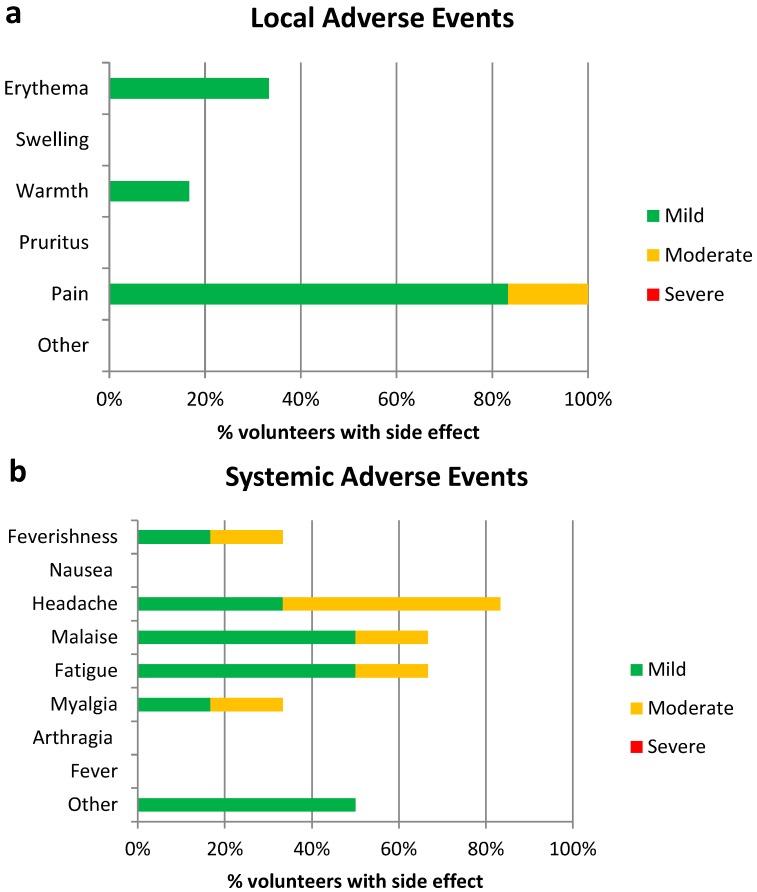
(**a**) Local and (**b**) systemic adverse events recorded after vaccination with a single dose of MVA-NP+M1.

There were no vaccine-related SAEs observed during the study. There were 29 AEs considered possibly, probably or definitely related to vaccination. All local and systemic solicited AEs were mild to moderate in nature (Figure 2) and resolved spontaneously within one to five days. All volunteers reported pain at the injection site (Figure 2a), and other local AEs reported included erythema and warmth at the injection site. None of the participants reported localized swelling or pruritus at the injection site. Headaches, followed by malaise and fatigue, were the most commonly reported systemic AEs (Figure 2b). “Other” systemic AEs were reported by 50% of volunteers, including pruritus (not at the injection site) and rhinorrhoea. One volunteer presented a transient and mild hypoalbuminemia which resolved within seven days. No documented fever was reported by any volunteers, whereas two reported subjective feverishness. Of all AEs reported, 72.41% (54.28–85.30, 95% CI) were mild and 27.59% (14.7–45.72, 95%CI) were moderate; 31.03% (17.28–49.23, 95%CI) were local and 68.97% (50.77–82.72, 95%CI) were systemic.

Moderate and severe, local and systemic solicited AEs reported in this trial were compared to the AEs reported from 24 subjects aged 18–50 in a previous study of MVA-NP+M1 manufactured on CEF at the same dose [10]. Differences between the proportions of moderate/severe AEs are summarized in Table 2 below.

### 3.3. T-Cell Responses

**Figure 3:**
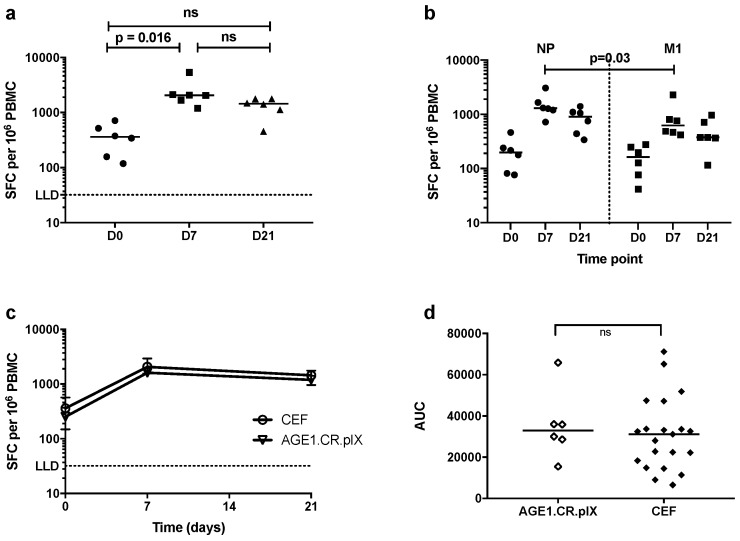
Ex vivo IFN-γ ELISpot responses to influenza antigens NP and M1 at baseline (D0), D7 and D21 post intramuscular vaccination with MVA-NP+M1 (1.5 × 10^8^ pfu) manufactured on AGE1.CR.pIX cell line. (**a**) Summed total response to NP and M1 from individual subjects. Comparisons across timepoints measured by Dunn’s multiple comparison tests; (**b**) responses to individual antigens, compared at each time point with two-tailed Wilcoxon signed-rank test; (**c**) median total responses to NP and M1 for individuals shown in (**a**) compared with those from a previous trial in which 21 volunteers received MVA-NP+M1 (1.5 × 10^8^ pfu) manufactured on CEF cell line [10]. Error bars are interquartile ranges; (**d**) area-under-curve (AUC) analysis of data in (**c**). The Mann–Whitney test was used to compare the two groups; ”ns”, not statistically significant (*p* > 0.05).

In addition to the primary objective of assessing the safety of MVA-NP+M1 manufactured on the AGE1.CR.pIX cell line, a secondary objective was to assess the cellular immune response to this vaccine. IFN-γ ELISpots were used to measure the frequency of antigen-specific T-cells at baseline (D0), one week (D7) and three weeks (D21) post vaccination (Figure 3a). Median IFN-γ ELISpots responses peaked at one week (2059, IQR 1563–567.1) post vaccination and persisted above baseline (360, IQR 148.3–567.1) to week three (1443, IQR 958–1766). Responses at D7 were significantly higher than at baseline (*p* = 0.016, Dunn’s multiple comparison test). Both antigens in the vector were immunogenic with higher responses to NP than M1 at both post vaccination time points (Figure 3b), despite no significant difference in response at D0 (*p* = 0.4, two-tailed Wilcoxon signed rank test). The peak response at D7 to NP was 1297 SFC (IQR 1080–2006) and that to M1 was 625.7 (IQR 457–1177; *p* = 0.03, two-tailed Wilcoxon signed-rank test), as shown in Figure 3b. 

Since this is the first use of MVA-NP+M1 manufactured on the AGE1.CR.pIX cell line, we compared IFN-γ ELISpot results in this study with those from a previous trial [10] in which volunteers received MVA-NP+M1 manufactured on CEF. ELISpot data at baseline, week one and week three were available from 21 volunteers in the previous trial within the same age group (18–50) and vaccine dose (1.5 × 10^8^ pfu) as this study. No difference at baseline was detected between the two cohorts (*p* = 0.6, two-tailed Mann–Whitney test), and the kinetics of the immune response induced were comparable (Figure 3c). AUC (incorporating D0, D7 and D21) was used as a metric to compare IFN-γ ELISpot responses between the six volunteers enrolled in this study and the 21 volunteers from the previous study (Figure 3d). No significant difference in AUC was observed between the two studies (two-tailed Mann–Whitney test), suggesting that there is no difference between the magnitudes of T-cell responses elicited by MVA-NP+M1 manufactured on the AGE1.CR.pIX cell line or CEF.

## 4. Discussion

In recent years, significant efforts have been made to address the highly variable and sub-optimal efficacy of currently available seasonal influenza vaccines. Adjuvanted, recombinant and high-dose vaccines have only modestly improved efficacy [18,19,20,21], especially in older adults where immunosenescence contributes to even lower efficacy rates, despite the excess risk that influenza presents due to the higher risk of serious complications from infection in this age group [7]. To date, none of these strategies have been able to overcome the risk of immunological escape due to accumulated antigenic drift mutations that result in mismatches between vaccine and circulating-virus strains. Different approaches towards the development of a universal influenza vaccine have been considered in the past few years, including vaccines targeting conserved antigens in the stalk domain of hemagglutinins, NP and M1, and most recently neuraminidases, but only a few have progressed into early-phase clinical trials [22,23]. 

Evidence from animal and human epidemiological studies during past influenza epidemics suggests that antigen-specific CD4^+^ and CD8^+^ T-cell responses can provide protective heterosubtypic immunity against influenza, which is especially important during seasonal antigenic drift, zoonotic infection or pandemics where there is no existing antibody response [24]. Although cross-reactive T-cells cannot prevent influenza infection, they have been associated with decreases in disease severity, duration and viral shedding [25,26]. 

We have previously established the safety and T-cell immunogenicity profiles of the poxvirus-vectored vaccine MVA-NP+M1 manufactured on CEF both in young and older adults. The vaccine has been designed to boost pre-existing cellular responses to NP and M1, conserved influenza antigens, which could provide broad-spectrum protection against influenza A viruses. The AGE1.CR.pIX avian cell line meets regulatory requirements and addresses the scalability limitations imposed by the CEF manufacturing process. 

In this study, we have shown that MVA-NP+M1 manufactured on the AGE1.CR.pIX duck cell line has a favourable and acceptable reactogenicity profile with mild and moderate self-limited adverse events and with similar cellular immunogenicity results compared to the previous batch manufactured on CEF. Moderate or severe pain at injection site was more frequently observed in participants receiving MVA-NP+M1 manufactured on CEF. However, this difference is more likely to be attributed to the higher volume given in order to achieve the same dose (1.15 mL in CEF vs 0.43 mL in AGE1.CR.pIX), rather than differences between the manufacturing processes in these cell lines. We recognize the small sample size as an important limitation of this study, which is not sufficiently powered to detect any statistically significant differences between the safety profiles of both cell lines. Nonetheless, this trial provides useful information on the reactogenicity of the AGE1.CR.pIX avian cell line where no serious adverse events related to the vaccine were reported.

A combined strategy to induce both T- and B-cell mediated immunity through co-administration of MVA-NP+M1 with a licensed inactivated influenza vaccine (IIV) has previously proven to be not only safe and feasible, but also able to improve humoral immune responses to IIV [11]. In order to assess the efficacy of this approach in older adults, we have designed a phase II, randomised, multi-centre, participant-blinded, placebo-controlled clinical endpoint trial to test efficacy of the co-administration of MVA-NP+M1 with IIV in adults vaccinated at the National Health Primary Care Services in England, UK (NCT03300362) [27]. Prior immune responses elicited by previous smallpox vaccination is a potential concern for the use of MVA-vectored vaccines in older age groups. The extent of the impact of anti-vector immunity on cellular responses to the proposed influenza antigens is yet to be clarified, but some evidence from pre-clinical and early phase clinical trials of different recombinant MVA vaccines seems to suggest it would be small [28,29,30]. Detailed immunophenotyping by flow cytometry, and assessment of the impact of pre-existing immunity, will be assessed in participants of the Phase IIb trial. 

## 5. Conclusions

MVA-NP+M1 manufactured on the novel AGE1.CR.pIX duck cell line is safe, well-tolerated and immunogenic. Both safety and immunogenicity profiles of MVA-NP+M1 manufactured on the AGE1.CR.pIX duck cell line are comparable to those of MVA-NP+M1 manufactured on CEF, which has been used in multiple previous trials. These positive results support clinical development of this vaccine into further clinical studies, the first of which will be a Phase IIb efficacy trial which will include co-administration of the new MVA-NP+M1 with a licensed quadrivalent inactivated vaccine in adults aged 65 and over.

## Figures and Tables

**Table 1:** Participant Demographics.

Variable	Number	Percentage
**Age**		
Median	32.5	
Interquartile range	24.25–48.5	
Sex		
Male	2	33.33
Female	4	66.67
Ethnicity		
White	5	83.33
Mixed (White and Black)	1	16.67

**Table 2:** Comparison between moderate and severe AEs in AGE1.CR.pIX vs CEF.

Adverse Event	Proportion of Moderate/Severe AEs–AGE1.CR.pIX (n = 6)	Proportion of Moderate/Severe AEs–CEF (n = 24)	Difference between Proportions	95% CI	*p* Value *
Pain at injection site	0.1667	0.5417	0.375	0.1228 to 0.8881	0.1755
Feverishness	0.1667	0.125	0.04167	−0.2208 to 0.5192	>0.9999
Arthralgia	0	0.0769	0.07692	−0.1128 to 0.5643	
Myalgia	0.1667	0.2917	0.125	−0.1465 to 0.6192	
Headache	0.5	0.25	0.25	−0.1726 to 0.6382	0.3287
Fatigue	0.1667	0.1667	0	−0.2669 to 0.4817	>0.9999
Nausea	0	0.125	0.1250	−0.08518 to 0.6169	
Malaise	0.1667	0.0833	0.08333	−0.1726 to 0.5569	0.5015

* Fisher’s Exact Test.

## Supplementary Materials

The following are available online at https://www.mdpi.com/2076-393X/7/1/33/s1, A phase I study to determine the safety and immunogenicity of the candidate influenza vaccine MVA-NP+M1 manufactured on the AGE1.CR.pIX novel avian cell line, in healthy adult volunteers.

## References

[1] Iuliano A.D., Roguski K.M., Chang H.H., Muscatello D.J., Palekar R., Tempia S., Cohen C., Gran J.M., Schanzer D., Cowling B.J. (2018). Estimates of global seasonal influenza-associated respiratory mortality: A modelling study. Lancet.

[2] Putri W., Muscatello D.J., Stockwell M.S., Newall A.T. (2018). Economic burden of seasonal influenza in the United States. Vaccine.

[3] Bardenheier B.H., Strikas R., Kempe A., Stokley S., Ellis J. (2007). Influenza vaccine supply, 2005–2006: Did we come up short?. BMC Health Serv Res..

[4] Belongia E.A., Simpson M.D., King J.P., Sundaram M.E., Kelley N.S., Osterholm M.T., McLean H.Q. (2016). Variable influenza vaccine effectiveness by subtype: A systematic review and meta-analysis of test-negative design studies. Lancet Infect. Dis..

[5] Wu N.C., Zost S.J., Thompson A.J., Oyen D., Nycholat C.M., McBride R., Paulson J.C., Hensley S.E., Wilson I.A. (2017). A structural explanation for the low effectiveness of the seasonal influenza H3N2 vaccine. PLoS Pathog..

[6] Demicheli V., Jefferson T., Di Pietrantonj C., Ferroni E., Thorning S., Thomas R.E., Rivetti A. (2018). Vaccines for preventing influenza in the elderly. Cochrane Database Syst. Rev..

[7] Haq K., McElhaney J.E. (2014). Immunosenescence: Influenza vaccination and the elderly. Curr. Opin. Immunol..

[8] Jefferson T., Rivetti D., Rivetti A., Rudin M., Di Pietrantonj C., Demicheli V. (2005). Efficacy and effectiveness of influenza vaccines in elderly people: A systematic review. Lancet.

[9] Lillie P.J., Berthoud T.K., Powell T.J., Lambe T., Mullarkey C., Spencer A.J., Hamill M., Peng Y., Blais M.E., Duncan C.J. (2012). Preliminary assessment of the efficacy of a T-cell-based influenza vaccine, MVA-NP+M1, in humans. Clin. Infect. Dis..

[10] Coughlan L., Sridhar S., Payne R., Edmans M., Milicic A., Venkatraman N., Lugonja B., Clifton L., Qi C., Folegatti P.M. (2018). Heterologous Two-Dose Vaccination with Simian Adenovirus and Poxvirus Vectors Elicits Long-Lasting Cellular Immunity to Influenza Virus A in Healthy Adults. EBioMedicine.

[11] Antrobus R.D., Berthoud T.K., Mullarkey C.E., Hoschler K., Coughlan L., Zambon M., Hill A.V., Gilbert S.C. (2014). Coadministration of seasonal influenza vaccine and MVA-NP+M1 simultaneously achieves potent humoral and cell-mediated responses. Mol. Ther..

[12] Antrobus R.D., Lillie P.J., Berthoud T.K., Spencer A.J., McLaren J.E., Ladell K., Lambe T., Milicic A., Price D.A., Hill A.V. (2012). A T cell-inducing influenza vaccine for the elderly: Safety and immunogenicity of MVA-NP+M1 in adults aged over 50 years. PLoS ONE.

[13] Berthoud T.K., Hamill M., Lillie P.J., Hwenda L., Collins K.A., Ewer K.J., Milicic A., Poyntz H.C., Lambe T., Fletcher H.A. (2011). Potent CD8+ T-cell immunogenicity in humans of a novel heterosubtypic influenza A vaccine, MVA-NP+M1. Clin. Infect. Dis..

[14] Jordan I., Vos A., Beilfuss S., Neubert A., Breul S., Sandig V. (2009). An avian cell line designed for production of highly attenuated viruses. Vaccine.

[15] Weissmahr R.N., Schüpbach J., Böni J. (1997). Reverse transcriptase activity in chicken embryo fibroblast culture supernatants is associated with particles containing endogenous avian retrovirus EAV-0 RNA. J. Virol..

[16] Shoyab M., Baluda M.A. (1975). Homology between avian oncornavirus RNAs and DNA from several avian species. J. Virol..

[17] Lohr V., Rath A., Genzel Y., Jordan I., Sandig V., Reichl U. (2009). New avian suspension cell lines provide production of influenza virus and MVA in serum-free media: Studies on growth, metabolism and virus propagation. Vaccine.

[18] DiazGranados C.A., Dunning A.J., Kimmel M., Kirby D., Treanor J., Collins A., Pollak R., Christoff J., Earl J., Landolfi V. (2014). Efficacy of high-dose versus standard-dose influenza vaccine in older adults. N. Engl. J. Med..

[19] Van Buynder P.G., Konrad S., Van Buynder J.L., Brodkin E., Krajden M., Ramler G., Bigham M. (2013). The comparative effectiveness of adjuvanted and unadjuvanted trivalent inactivated influenza vaccine (TIV) in the elderly. Vaccine.

[20] Mannino S., Villa M., Apolone G., Weiss N.S., Groth N., Aquino I., Boldori L., Caramaschi F., Gattinoni A., Malchiodi G. (2012). Effectiveness of adjuvanted influenza vaccination in elderly subjects in northern Italy. Am. J. Epidemiol..

[21] Dunkle L.M., Izikson R., Patriarca P., Goldenthal K.L., Muse D., Callahan J., Cox M.M.J., Team P.S.C.S. (2017). Efficacy of Recombinant Influenza Vaccine in Adults 50 Years of Age or Older. N. Engl. J. Med..

[22] Rajao D.S., Perez D.R. (2018). Universal Vaccines and Vaccine Platforms to Protect against Influenza Viruses in Humans and Agriculture. Front. Microbiol..

[23] Kumar A., Meldgaard T.S., Bertholet S. (2018). Novel Platforms for the Development of a Universal Influenza Vaccine. Front. Immunol..

[24] Clemens E.B., van de Sandt C., Wong S.S., Wakim L.M., Valkenburg S.A. (2018). Harnessing the Power of T Cells: The Promising Hope for a Universal Influenza Vaccine. Vaccines.

[25] Hayward A.C., Wang L., Goonetilleke N., Fragaszy E.B., Bermingham A., Copas A., Dukes O., Millett E.R., Nazareth I., Nguyen-Van-Tam J.S. (2015). Natural T Cell-mediated Protection against Seasonal and Pandemic Influenza. Results of the Flu Watch Cohort Study. Am. J. Respir. Crit. Care Med..

[26] Sridhar S., Begom S., Bermingham A., Hoschler K., Adamson W., Carman W., Bean T., Barclay W., Deeks J.J., Lalvani A. (2013). Cellular immune correlates of protection against symptomatic pandemic influenza. Nat. Med..

[27] Clinicaltrials.gov NCT03300362. Improved Novel VaccIne CombinaTion InflUenza Study (INVICTUS). NCT03300362.

[28] Cottingham M.G., Carroll M.W. (2013). Recombinant MVA vaccines: Dispelling the myths. Vaccine.

[29] Ramírez J.C., Gherardi M.M., Rodríguez D., Esteban M. (2000). Attenuated Modified Vaccinia Virus Ankara Can Be Used as an Immunizing Agent under Conditions of Preexisting Immunity to the Vector. J. Virol..

[30] Harrop R., Connolly N., Redchenko I., Valle J., Saunders M., Ryan M.G., Myers K.A., Drury N., Kingsman S.M., Hawkins R.E. (2006). Vaccination of Colorectal Cancer Patients with Modified Vaccinia Ankara Delivering the Tumor Antigen 5T4 (TroVax) Induces Immune Responses which Correlate with Disease Control: A Phase I/II Trial. Clin. Cancer Res..

