# Supplementary material for: Safety and Immunogenicity of the Heterosubtypic Influenza A Vaccine MVA-NP+M1 Manufactured on the AGE1.CR.pIX Avian Cell Line
Source: Vaccines (Basel). 2019 Mar 22;7(1):33. doi: 10.3390/vaccines7010033 (PMC6466103; doi:10.3390/vaccines7010033)
Supplement: Supplementary file 1 [file vaccines-07-00033-s001.pdf]

A phase I study to determine the safety and immunogenicity of the candidate influenza vaccine MVA-NP+M1 manufactured on the AGE1.CR.pIX novel avian cell line, in healthy adult volunteers

Study Reference: FLU008

Protocol Version: 1.0

Date: 14th March 2017

Chief Investigator: Professor Adrian Hill

Sponsor: Vaccitech

REC Number: 17/SC/0288

EudraCT Number: 2017-001104-30

Conflict of Interest

According to the Declaration of Helsinki, 2008, I have read this protocol, and declare the following conflict of interest

Prof Adrian Hill is scientific co-founder of the spin-out Company and sponsoring institution named Vaccitech

		24/04/2017
**Chief Investigator**	**Investigator Signature**	**Date**
**Prof. Adrian Hill**		

Details:____

**Full study title:**	**A phase I study to determine the safety and immunogenicity of the candidate influenza vaccine MVA-NP+M1, manufactured on the AGE1.CR.pIX novel avian cell line, in healthy adult volunteers**
	Study Code: **FLU008**
	EudraCT Number: **2017-001104-30**
	IRAS Project ID: **225916**
**Chief Investigator**	**Prof. Adrian V S Hill**<br>Centre for Clinical Vaccinology and Tropical Medicine<br>University of Oxford,<br>Churchill Hospital, Old Road, Headington<br>Oxford, OX3 7LE<br>Email: adrian.hill@ndm.ox.ac.uk
**Trial Sites**	**Centre for Clinical Vaccinology and Tropical Medicine**<br>Churchill Hospital, Old Road, Headington<br>Oxford, OX3 7LE<br>Tel: 01865 857417<br>Fax: 01865 857471
**Sponsoring Institution**	**Vaccitech Limited**<br>The Weston Library<br>Broad Street<br>Oxford<br>Oxfordshire<br>OX31 3BG
**Local Safety Monitor (Chairman of Local Safety Committee)**	**Dr Brian Angus**<br>Centre for Clinical Vaccinology and Tropical Medicine<br>Churchill Hospital, Old Road, Headington<br>Oxford, OX3 7LE<br>Tel: 01865 220289<br>Email: brian.angus@ndm.ox.ac.uk

Confidentiality Statement

This document contains confidential information that must not be disclosed to anyone other than the Sponsor, the Investigator Team, the Regulatory authorities and members of the Independent Ethics Committee. This information cannot be used for any purpose other than the evaluation or conduct of the clinical investigation without the prior written consent of Professor Adrian Hill.

Statement of Compliance

The trial will be conducted in compliance with the protocol, International Conference on Harmonisation Good Clinical Practice Guideline E6 (R1) (ICH-GCP) and the applicable regulatory requirements.

Investigator Agreement

“I have read this protocol and agree to abide by all provisions set forth therein.
I agree to comply with the principles of the International Conference on Harmonisation Tripartite Guideline on Good Clinical Practice.”

Chief Investigator: ______________________________ Date: 24/04/2017
Adrian Hill
Professor of Human Genetics, University of Oxford
Centre for Clinical Vaccinology and Tropical Medicine

Modification History

**Version**	**Date**	**Author(s)**	**Modifications**

TABLE OF CONTENTS

TABLE OF CONTENTS

1. SYNOPSIS 11
2. ABBREVIATIONS 13
3. BACKGROUND AND RATIONALE 15
3.1 The need for a new vaccine against influenza 15
3.2 Previous experience with MVA vectored vaccines. 16
3.3 The novel avian cell line AGE1.CR.pIX 17
3.4 The selection of NP+M1 as an insert for a viral vectored vaccine 18
3.5 Pre-Clinical Studies 19
3.6 Previous clinical experience 19
3.7 Rationale 21
3.8 Vaccine development strategy 22
4. Trial design 23
4.1 Study group 23
4.2 First volunteers 23
4.3 Duration of study 23
4.4 Definition of Start and End of Trial 23
4.5 Potential Risks for volunteers 24
4.6 Known Potential Benefits 24
5. OBJECTIVES AND ENDPOINTS 25
5.1 Primary Objectives 25
5.1.1 Primary Outcome Measures 25
5.2 Secondary Objectives 25
5.2.1 Secondary Outcome Measures 25
6. INVESTIGATIONAL PRODUCT 27
6.1 Manufacturing and presentation 27
6.2 Supply 27
6.3 Storage 27

6.4 Administration 27

6.5 Rationale for selected vaccine dose 27

6.6 Minimising environmental contamination with genetically modified organisms (GMO) 28

7 RECRUITMENT AND WITHDRAWAL OF TRIAL VOLUNTEERS 29

7.1 Identification of trial volunteers 29

7.2 Informed consent 29

7.3 Inclusion and exclusion criteria 30

7.3.1 Inclusion Criteria 30

7.3.2 Exclusion Criteria 31

7.3.3 Effective contraception for female volunteers 32

7.3.4 Prevention of 'Over Volunteering' 32

7.3.5 Criteria for postponement of vaccination 32

7.3.6 Withdrawal of Volunteers 33

7.4 Compliance with Dosing Regime 33

7.5 Pregnancy 34

8 TREATMENT OF TRIAL VOLUNTEERS 35

8.1 Study procedures 35

8.2 Observations 35

8.3 Blood tests and urinalysis 35

8.4 Study visits 36

8.4.1 Screening visit 36

8.4.2 Day 0: Enrolment and vaccination visit 37

9 ASSESSMENT OF SAFETY 40

9.1 Interim Safety Reviews 40

9.2 Definitions 40

9.2.1 Adverse Event (AE) 40

9.2.2 Adverse Reaction (AR) 40

9.2.3 Unexpected Adverse Reaction 40

9.2.4 Serious Adverse Event (SAE) 41

9.2.5 Serious Adverse Reaction (SAR) 41

9.2.6 Suspected Unexpected Serious Adverse Reaction (SUSAR) 41
9.3 Foreseeable Adverse Reactions: 41
9.4 Expected Serious Adverse Events 42
9.5 Causality Assessment 42
9.6 Reporting Procedures for All Adverse Events 43
9.6.1 Reporting Procedures for Serious AEs 44
9.6.2 Reporting Procedures for SUSARS 44
9.6.3 Development Safety Update Report 44
9.7 Assessment of severity 44
9.8 Procedures to be followed in the event of abnormal findings 46
9.9 Local Safety Committee 46
9.9.1 Safety Profile Review 47
9.10 Safety Holding Rules 47
10 STATISTICS 48
11. QUALITY CONTROL AND QUALITY ASSURANCE PROCEDURES 50
11.1 Investigator procedures 50
11.2 Monitoring 50
11.3 Modification to protocol 50
11.4 Protocol deviation 50
11.5 Audit & inspection 50
11.6 Serious Breaches 51
11.7 Trial Progress 51
11.8 Publication Policy 51
12. ETHICS AND REGULATORY CONSIDERATIONS 53
12.1 Declaration of Helsinki 53
12.2 Guidelines for Good Clinical Practice 53
12.3 Informed Consent 53
12.4 Approvals 53
12.5 Volunteer Confidentiality 53
13. DATA HANDLING AND RECORD KEEPING 54

13.1 Data Handling 54
13.2 Record Keeping 54
13.3 Source Data and Case Report Forms (CRFs) 54
13.4 Data Protection 54
14 FINANCING AND INSURANCE 55
14.2 Financing 55
14.3 Insurance 55
14.4 Compensation 55

1. SYNOPSIS

**Title**	A phase I study to determine the safety and immunogenicity of the candidate influenza vaccine MVA-NP+M1, manufactured on the AGE1.CR.pIX novel avian cell line, in healthy adult volunteers.
**Trial Centres**	Centre for Clinical Vaccinology & Tropical Medicine, University of Oxford, Churchill Hospital, Old Road, Headington, Oxford, OX3 7LE.
**Trial Identifier**	FLU008
**Clinical Phase**	I
**Design**	Open label, phase I clinical trial.
**Target Population**	Healthy adults aged 18 to 50
**Sample Size**	Total: 6

Group	Dose
Group 1 (n=6)<br>Aged 18-50	1.5 x10^8 pfu (converted from $TCID_{50}$) MVA-NP+M1 alone

**Follow-up duration**	4 weeks
**Planned Trial Period**	Q3 2017 – Q4 2017
**Primary Objectives**	To assess the safety of MVA-NP+M1, manufactured on the AGE1.CR.pIX novel avian cell line.
**Secondary Objectives**	To assess the cellular immune response generated by the MVA-NP+M1, manufactured on the AGE1.CR.pIX novel avian cell line.
**Investigational products**	MVA-NP+M1
**Form**	Liquid

**Route of Administration**	Intramuscular (IM) injection into the Deltoid
**Dose per Administration**	1.5 x10^8 pfu (converted from $TCID_{50}$)

2. ABBREVIATIONS

**AE**	Adverse event
**CCVTM**	Centre for Clinical Vaccinology and Tropical Medicine, Oxford
**CBF**	Clinical BioManufacturing Facility
**CEF**	Chick embryo fibroblast
**CI**	Chief Investigator
**CRF**	Case Report Form or Clinical Research Facility
**DSUR**	Development Safety Update Report
**ELISPOT**	Enzyme-linked immunospot
**GCP**	Good Clinical Practice
**GMO**	Genetically modified organism
**GP**	General Practitioner
**HCG**	Human Chorionic Gonadotrophin
**HBV**	Hepatitis B virus
**HCV**	Hepatitis C virus
**HIV**	Human immunodeficiency virus
**IB**	Investigator Brochure
**ICH**	International Conference on Harmonisation
**ID**	Intradermal
**IFNγ**	Interferon gamma
**IM**	Intramuscular
**IMP**	Investigational Medicinal Product
**IMP-D**	Investigational Medicinal Product Dossier
**IV**	Intravenous
**LSM**	Local safety monitor
**MHRA**	Medicines and Healthcare Products Regulatory Agency
**MVA**	Modified vaccinia virus Ankara
**NHS**	National Health Service
**PBMC**	Peripheral blood mononuclear cell
**pfu**	Plaque forming unit
**QP**	Qualified Person
**REC**	Research Ethics Committee
**SAE**	Serious adverse event
**SOP**	Standard Operating Procedure

**SUSAR**	Suspected unexpected serious adverse reaction
**$TCID_{50}$**	Median tissue culture infective dose
**WHO**	World Health Organisation

3. BACKGROUND AND RATIONALE

3.1 The need for a new vaccine against influenza

Influenza is an orthomyxovirus and encodes a segmented RNA genome. Influenza is divided into 3 groups- A, B and C. Most seasonal influenza and all known pandemics are caused by Influenza A, whilst B and C cause low-levels disease and sporadic outbreaks (1). Influenza A is subdivided further on the basis of hemagglutinin (H or HA) and neuraminidase (N or NA) activity- for example, H1N1 subtype. There are at least 16 different types of HA and 9 of NA. These proteins are found as spiked surface projections on the Influenza A virus (2). Other genes encode proteins vital for structure, reproduction and virulence including nucleoprotein (NP), Matrix 1 (M1), M2 (ion pore), NS1, NS2, PA, PB1, PB1-F2 and PB2, which are found within the envelope (1, 2).

Seasonal influenza has a huge annual global impact accounting for an estimated 1 billion illnesses and 250,000 – 500,000 deaths (3) with an economic cost of annual seasonal influenza estimated to be $87.1 billion in the US alone (4). Influenza pandemics also occasionally occur, leading to increased significant health and economic burden (5). The unpredictable risk of sporadic outbreaks of human infections with avian influenza (H5N1), which could trigger a new pandemic if the virus acquires the ability to transmit from person to person, (6) makes influenza a major global public health issue. Young children, the elderly, pregnant women and those with significant co-morbidities such as asthma, chronic respiratory disease and immunosuppressive conditions remain at highest risk of influenza infection.

Vaccination remains the most cost-effective strategy available to combat influenza. The currently available influenza vaccines work in part by inducing strain-specific antibodies against the highly polymorphic surface proteins (hemagglutinin, neuraminidase) of the influenza virus. Inactivated or live vaccines are made up of proteins or live viruses covering four influenza virus strains (H1N1, H3N2 and two strains of influenza B) which are predicted to circulate in the population in the upcoming influenza season. As the circulating virus strains change, influenza vaccines need to be reformulated annually to match new strains arising from genetic drifts on the surface proteins of these seasonal viruses. These surface proteins, to which vaccine-induced antibodies are targeted, are highly polymorphic. There is little protection against strains of a new subtype and limited protection even across strains within the same subtype. Approximately once every 20 years, vaccine efficacy happens to be much lower than expected due to significant antigenic drifts away from the vaccine strain (7). This need for constant redesign and remanufacture increases the vaccine's cost, places limitations on supply (8) and critically delays vaccine production. When new strains arise, the HA and NA sequences need to be identified and incorporated into the new vaccine, leaving large populations susceptible to infection and illness from the new strains. However, not only is the efficacy of current vaccines limited in the face of antigenic mismatch

between circulating strains and those in the vaccine but it is also substantially reduced in elderly groups. Vaccination in older adults prevents laboratory-confirmed influenza in only 30–40% compared to the 70–90% protection found in young adults (9). Thus, there is an unmet need for improved vaccination strategies that can provide protection against a broader spectrum of virus strains particularly for the elderly.

Where individuals exposed to a newly arisen influenza virus strain lack protective neutralising antibodies, cross-reactive T-cells against conserved internal antigens of influenza have been shown to be associated with limited viral shedding, reduced duration of symptoms and minimised severity of symptomatic illness (10, 11). Thus, an influenza vaccine capable of inducing protective T cell responses against conserved internal antigens could provide lasting immunity against not only human seasonal influenza, but also subtypes currently found in avian species or swine (which could potentially cause new pandemics). Since adults have been primed by previous influenza exposure, a vaccine expressing conserved internal antigens of influenza (such as NP and M1) could be used to boost cross-reactive T-cell responses to protective levels, providing durable broad immunity to all subtypes of influenza A.

Recombinant viral vectored vaccines have been used in humans to induce high frequencies of CD4+ and CD8+ T cell responses to a wide range of antigens. One such recombinant viral vector is the Modified Vaccinia Ankara (MVA) which has been used to generate strong T-cell responses to a wide range of antigens, including antigens from plasmodium, tuberculosis, hepatitis C, HIV and influenza. The MVA-NP+M1 is a recombinant replication-deficient MVA vector expressing the influenza antigens NP and M1 as a fusion protein (12).

Vaccination with MVA-NP+M1 leads to a rapid increase of influenza-specific cross-reactive IFN-γ-secreting effector T cells, which are maintained at protective levels over a prolonged period(12). In the older age groups, MVA-NP+M1 can boost pre-existing levels of influenza-specific T-cells and maintain them for up to six months post-vaccination (13). The MVA-NP+M1 combined with a licensed inactivated influenza vaccine induced influenza-specific T-cells and increased the magnitude and breadth of the antibody response induced by the inactivated influenza vaccine (14). Furthermore, in a Phase IIa challenge study of MVA-NP+M1 alone, vaccinated individuals experimentally challenged with live influenza virus had less severe symptoms and significantly shorter duration of viral shedding compared to unvaccinated controls (15). Taken together, these studies demonstrate the efficacy of MVA-NP+M1 in limiting the severity of influenza illness and its higher immunogenicity in older adults when combined with a licensed inactivated seasonal influenza vaccine.

3.2 Previous experience with MVA vectored vaccines.

MVA is an attractive candidate orthopox vaccine vector for safety and immunogenicity reasons. The successful worldwide eradication of smallpox using vaccination with the vaccinia virus highlighted it as a candidate vaccine vector. Although millions of humans have

been vaccinated with conventional replication-competent vaccinia virus, its small but definite risk to both researchers and future patients led to the development of several attenuated strains of vaccinia during early smallpox eradication strategies and more recently as well. The host-range restricted MVA particularly proved to be extremely attenuated compared to other vaccinia viruses.

MVA was originally derived from the vaccinia strain Ankara by over 500 serial passages in primary chicken embryo fibroblasts (CEF cells). MVA has six major genomic deletions compared to the parental Ankara genome and is severely compromised in its ability to replicate in mammalian cells. No replication has been documented in non-transformed mammalian cells. The viral genome has been proven to be stable through a large series of passages in chicken embryo fibroblasts (16). MVA also showed no cytopathic effect or plaque formation in cells of human origin. In irradiated mice, it did not elicit any morbidity or lethality even when administered at high doses intra-cerebrally, indicating its safety even in immuno-compromised organisms (16).

MVA has an excellent safety record and it has been administered to approximately 120,000 people during a smallpox eradication campaign in a field study in Germany in the 1970s (17). MVA is currently in development as a vector for multiple diseases including HIV-1, (18, 19) tuberculosis, (20) HCV (21), influenza (12) and melanoma (22). The MVA vectored vaccines developed at the University of Oxford have been administered to over 4570 individuals including infants, young children, elderly adults, HIV-infected adults and children and patients with cancer in Europe and Africa without any safety concerns. Clinical studies have shown intramuscular administration, as compared to intradermal, to be associated with fewer and short-lived local AEs and no reduction in immunogenicity (23).

3.3 The novel avian cell line AGE1.CR.pIX

There are a number of disadvantages to using CEF cells for the production of MVA vectored vaccines including limited scalability of the process, possible shortage of material in case of an avian pandemic and occasional difficulties with bacterial contamination of embryonated eggs. Therefore, the MVA-NP+M1 for use in this study is being produced in the novel immortalised duck retinal cell line AGE1.CR.pIX grown in a serum-free medium. This cell line was designed according to the current regulatory requirements and was optimized to address the scalability limitations imposed by the CEF manufacturing process.

The avian cell line AGE1.CR (CR) was created by immortalizing cells from duck retina with adenovirus type 5 E1 genes. Ducks as opposed to chickens carry significantly fewer endogenous retroviral inserts, meaning the novel cell line is also safer than previous attempts to use cell lines derived from spontaneous immortalization of embryonic chicken cells. The CR line was further modified for constitutive expression of the adenoviral pIX gene (to obtain CR.pIX) that encodes for a structural protein involved in capsid stabilization of cognate adenovirus. CR and CR.pIX cell lines proliferate in suspension in serum-free media.

Exceptional genetic stability was demonstrated for more than 90 passages. The cells can be productively infected with viruses of diverse families including different influenza strains and MVA (24).

The AGE1.CR.pIX avian cell line typically proliferates with an indefinite life span in suspension in serum-free medium with zero or low protein content. Immortalization is achieved by transfection of the E1A and E1B genes from the human adenovirus serotype 5. The cell lines are adapted to proliferation in suspension in media free of animal derived components. Both in adherent monolayers and in suspension culture the cells are highly permissive for MVA, surpassing yields obtained with primary chicken fibroblasts (25).

3.4 The selection of NP+M1 as an insert for a viral vectored vaccine

The inner proteins of the influenza virus are more conserved and less prone to antigenic drifts compared to the external surface glycoproteins. There are two reasons for selecting NP and M1 as a target for T-cell inducing vaccines. Firstly, there is very little polymorphism of NP and M1 across influenza A isolates. NP is 92% identical between H3N2 and H1N1 strains, and 91% identical between H3N2 and H5N1 strains. M1 is 95% identical between H3N2 and H1N1 strains, and 93% identical between H3N2 and H5N1 strains. This low level of variation appears to allow strong T cell cross-reactivity. H3N2-derived antigen sequences (from A/Panama/2007/99) have been included as this is the subtype to which most people will have memory T cell responses. In the local population more than 70% of individuals generate a T cell response to these two antigens (26). Secondly, analysis of T-cell response to all the proteins of influenza have shown that the T-cell response to NP and M1 is the strongest and 80% of individuals have responses to these two proteins (Figure 1). Furthermore, recent studies have shown that T-cells specific to M1 and NP are associated with protecting individuals against influenza by limiting viral shedding, reduced duration of symptoms and minimising severity of symptomatic illness (10, 11).

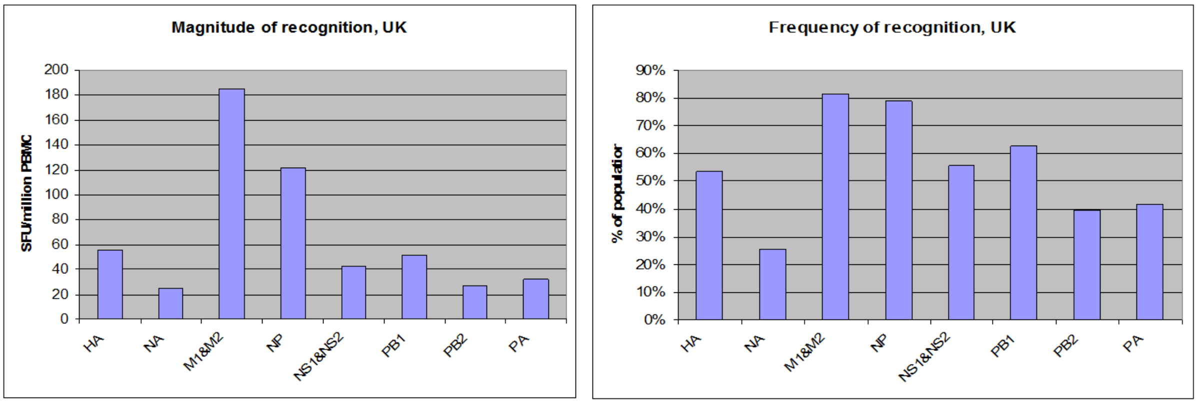

Figure 1. Frequency and magnitude of T cell responses to influenza antigens in UK adults. Responses to M1 and M2 were tested together, but in the small number of volunteers where these were separated the response was predominantly to M1 rather than M2.

HA	hemagglutinin	NS1	non-structural protein 1
NA	neuraminidase	NS2	non-structural protein 2
M1	matrix protein 1	PB1	polymerase subunit B1
M2	matrix protein 2	PB2	polymerase subunit B2
NP	nucleoprotein	PA	polymerase subunit A

3.5 Pre-Clinical Studies

The MVA viral vector manufactured using the AGE1.CR.pIX avian cell line has been previously used with a different insert for an Ebola preventive vaccine (MVA-EBOZ). A GLP-compliant non-clinical toxicology study was conducted to evaluate the local and systemic toxicity of the MVA85A vaccine manufactured using the AGE1.CR.pIX® avian cell line. The MVA85A was used as a surrogate test article to support the use of a MVA-EBOZ vaccine produced in the AGE1.CR.pIX® avian cell line and to demonstrate the safety of the vaccine for the first in human trial. The manufacturing process to produce MVA-EBOZ was derived from that used for MVA85A. The toxicology study performed on BALB/c mice revealed that repeated administration of MVA85A had no effect on mortality, cage side observations or bodyweights. Mice gained weight and had normal food consumption during the study. Clinical observations, inoculation site reactogenicity, clinical chemistry, clinical haematology, gross necropsy, organ weights and histopathology indicated no overt toxicity related to MVA85A vaccine administration (see IB for MVA-EBO Z).

3.6 Previous clinical experience

This is the first-in-human trial of MVA-NP+M1 produced using the AGE1.CR.pIX immortal avian cell line. However, MVA-NP+M1 manufactured using CEF cells has been administered to 145 adults in six clinical trials (5 phase I trials and 1 Phase IIa challenge study). The vaccine has been shown to have a good safety profile with no vaccine related serious adverse events during these trials. The vaccine was safe and boosted T-cell responses as expected when administered to healthy adults. A dose dependent increase in adverse events was observed and a dose of 1.5 $x10^8$ pfu was found to be the optimal balance between immunogenicity and reactogenicity (12). It has also been given at the same time as the standard seasonal influenza vaccine (FLU003) and has been shown to have a good safety profile. MVA NP+M1 has already been shown to provide protection against viral shedding and severity of symptoms in a Phase IIa influenza challenge study (FLU002). In FLU002, fewer vaccinated volunteers developed influenza symptoms than the unvaccinated

volunteers and there was a statistically significant reduction in duration of virus shedding in vaccinated volunteers (15).

MVA-NP+M1 has been administered to 145 individuals across a range of doses and via both the intramuscular and intradermal routes, as shown in Table 1. Figure 2 demonstrates the immunogenicity (as determined by interferon-gamma ELISpot) of MVA-NP+M1 in older adults (aged 50+) receiving a dose of 1.5 x10^8 pfu.

Table 1. Previous MVA-NP+M1 trials

Country	Study	Vaccine	Age	Route	Dose of MVA-NP+M1	Number of volunteers
UK	FLU001	MVA-NP+M1	18-50	ID	5 x10^7 pfu	12
		MVA-NP+M1	18-50	IM	5 x10^7 pfu	8
		MVA-NP+M1	18-50	IM	2.5 x10^8 pfu	8
		MVA-NP+M1	50-59	IM	1.5 x10^8 pfu	10
		MVA-NP+M1	60-69	IM	1.5 x10^8 pfu	10
		MVA-NP+M1	70+	IM	1.5 x10^8 pfu	10
UK	FLU002	MVA-NP+M1	18-50	IM	1.5 x10^8 pfu	15
UK	FLU003	MVA-NP+M1 *(together with seasonal influenza vaccine)*	50+	IM	1.5 x10^8 pfu	9
UK	FLU004	ChAdOx1-NP+M1/MVA-NP+M1 *(7-14 weeks apart)*	18-50	IM	1.5 x10^8 pfu	3
UK	FLU005	ChAdOx1-NP+M1 / MVA-NP+M1 *(8 weeks apart)*	18-50	IM	1.5 x10^8 pfu	12
		ChAdOx1-NP+M1 / MVA-NP+M1 *(52 weeks apart)*	18-50	IM	1.5 x10^8 pfu	8
		MVA-NP+M1 / ChAdOx1-NP+M1 *(8 weeks apart)*	18-50	IM	1.5 x10^8 pfu	13
		MVA-NP+M1 / ChAdOx1-NP+M1 *(52 weeks apart)*	18-50	IM	1.5 x10^8 pfu	12
		ChAdOx1-NP+M1 / MVA-NP+M1 *(8 weeks apart)*	>50+	IM	1.5 x10^8 pfu	12
UK	FLU006	MVA-NP+M1 (*co- administered with seasonal influenza vaccine - Viroflu®*	18-50	IM	1.5 x10^8 pfu	3

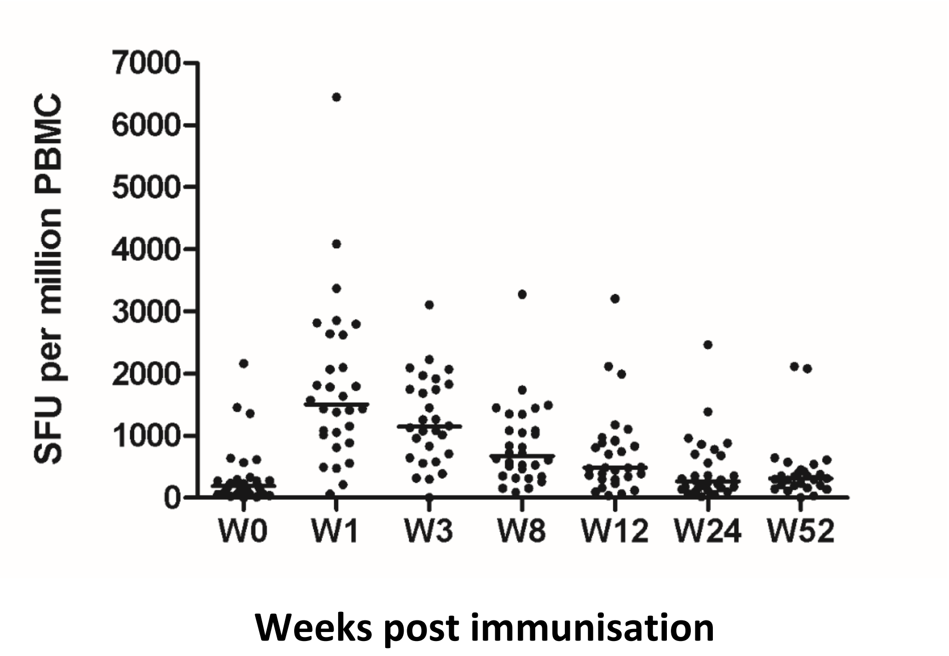

Figure 2: Immunogenicity of MVA-NP+M1 in older adults (FLU001)

The EBL04, a phase I Ebola vaccine trial, was the first administration of MVA-EBO Z in humans and the first trial of any MVA bio-manufactured on an immortalised duck retinal cell line, instead of the standard chicken embryo fibroblast primary cells. The trial enrolled 38 healthy adult volunteers in the UK and follow up is still ongoing. To this date, no significant safety concerns have been reported. A few volunteers reported moderate local and systemic adverse events. One volunteer reported severe local erythema on day 4 post-vaccination. One volunteer reported severe fevers associated with severe fatigue and malaise. MVA-EBO Z was generally well tolerated, causing mild-to-moderate short-lived local and systemic adverse events. There have not been any vaccine related SAEs until this date (see development safety update report for EBL04)

3.7 Rationale

Vaccination remains the most cost-effective strategy available to combat influenza. Current influenza vaccines work by inducing strain-specific antibodies against the highly polymorphic surface proteins of the influenza virus. The need for constant redesign and remanufacture increases the vaccine's cost, places limitations on supply and critically delays vaccine production when new strains arise. Thus, there is a major demand for improved vaccination strategies that can provide protection against a broad spectrum of virus strains.

The elderly are at higher risk of severe disease and vaccination in older adults has poor efficacy, preventing only 30-40% of laboratory-confirmed influenza. Previous MVA-NP+M1 trials have shown satisfactory immunogenicity across different age groups with tolerable reactogenicity. A phase IIa influenza challenge study has shown the MVA-NP+M1 is able to

provide protection against viral shedding and to reduce severity of influenza symptoms. The MVA-NP+M1 produced in the novel immortalised duck retinal cell line AGE1.CR.pIX addresses the scalability issues of CEF produced vaccines, optimising vaccine production processes.

3.8 Vaccine development strategy

The MVA-NP+M1 is a Modified Vaccinia Ankara vectored vaccine, manufactured on the novel avian cell line AGE1.CR.pIX, expressing the conserved influenza A nucleoprotein and matrix protein. T-cell responses specific to M1 and NP, elicited by this viral vectored vaccine, are associated with protecting individuals in a human influenza challenge study by limiting viral shedding, reduced duration of symptoms and minimising severity of symptomatic illness. Previous clinical trials have shown the vaccine is satisfactory safe and immunogenic across different age groups.

The trial aims to contribute towards the development of a more efficacious vaccine capable of providing protection against a broad spectrum of influenza A virus strains with better immune responses in those at higher risk of severe influenza disease.

The MVA-NP+M1 produced in immortalised avian cell line will be used in a subsequent phase IIb clinical trial involving 2,000 volunteers, providing there are no safety concerns identified during this phase I trial.

Taking together the previous safe use of MVA-NP+M1 manufactured in CEF cells and MVA-EBOZ manufactured in AGE1.CR.pIX, there is no reason to suspect that the adverse event profile of MVA-NP+M1 manufactured in AGE1.CR.pIX will differ from that of MVA-NP+M1 manufactured in CEF cells. Conducted prior to initiating a phase IIb study in 2000 individuals in which 1000 individuals will receive MVA-NP+M1, this small phase I bridging study will allow the investigators to confirm that there is no difference in the safety profile of the vaccine as a result of a change in the cells used for production.

4. TRIAL DESIGN

This is a first in human, phase I, open label study of the MVA viral vector (produced in the novel immortalised duck retinal cell line AGE1.CR.pIX) expressing the influenza antigens NP and M1 as a fusion protein, in healthy adult volunteers. MVA-NP+M1 will be given alone intramuscularly as a single dose.

There will be 1 study group and a total of 6 volunteers will be enrolled (table 2). Staggered enrolment will apply for the first three volunteers within the group. Volunteers will be allocated by selecting eligible volunteers for enrolment in the order in which they were deemed eligible following screening.

4.1 Study group

Table 2. Study group.

Group	Dose	Route
Group 1 (n=6)<br>Aged 18-50	1.5 $x10^8$ pfu (converted from $TCID_{50}$) MVA-NP+M1	IM

4.2 First volunteers

The first volunteer will be vaccinated alone and then reviewed 48 hours following vaccination. If there are no safety concerns, another two volunteers may be vaccinated at least one hour apart from each other, and reviewed in a further 48 hours. Providing there are no safety concerns as assessed by the Chief Investigator (CI) and LSM (Local Safety Monitor), the remaining 3 volunteers in the group may be vaccinated. An independent safety review will be carried out by the LSM (chair of the Local Safety Committee) after the first 3 volunteers in the group have been vaccinated. This review will include the results of safety blood tests at day 7 post vaccination.

4.3 Duration of study

The total duration of the study will be 4 weeks from the day of enrolment for all volunteers.

4.4 Definition of Start and End of Trial

The start of the trial is defined as the date of the first vaccination of the first volunteer. The end of the trial is the date of the last visit of the last volunteer.

4.5 Potential Risks for volunteers

The potential risk to participants is considered as low. The potential risks are those associated with phlebotomy and vaccination.

Phlebotomy: The maximum volume of blood drawn over the study period (approximately 180 mL) should not compromise these otherwise healthy volunteers. There may be minor bruising, local tenderness or pre-syncopal symptoms associated with venepuncture, which will not be documented as AEs if they occur.

Vaccination: Vaccination usually precipitates a local inflammatory reaction. Potential expected risks from vaccination include local effects such as pain, redness, warmth, swelling, scaling, tenderness or itching. Systemic reactions that could potentially occur following immunisation with a recombinant MVA vaccine include a flu-like illness with feverishness, fatigue, malaise, arthralgia, myalgia and headache. As with any vaccine, Guillain-Barré syndrome or immune-mediated reactions that can lead to organ damage may occur, but this should be extremely rare. Serious allergic reactions including anaphylaxis could also occur and for this reason volunteers will be vaccinated in a clinical area where Advanced Life Support trained physicians, equipment and drugs are immediately available for the management of any serious adverse reactions (SAR).

4.6 Known Potential Benefits

Volunteers will not benefit directly from participation in this study. However, it is hoped that the information gained from this study will contribute to the development of a safe and effective influenza vaccine regimen. The only benefits for participants would be information about their general health status.

5. OBJECTIVES AND ENDPOINTS

The number of volunteers has been chosen to generate adequate safety and immunogenicity data to meet these objectives

5.1 Primary Objectives

To assess the safety and reactogenicity of the candidate vaccine MVA-NP+M1 manufactured on the AGE1.CR.pIX avian cell line.

5.1.1 Primary Outcome Measures

The specific endpoints for safety and reactogenicity will be actively and passively collected data on adverse events

The following parameters will be assessed for the study group

- Occurrence of solicited local reactogenicity signs and symptoms for 7 days following the vaccination
- Occurrence of solicited systemic reactogenicity signs and symptoms for 7 days following the vaccination
- Occurrence of unsolicited adverse events for 28 days following the vaccination
- Change from baseline for safety laboratory measures
- Occurrence of serious adverse events during the whole study duration

Volunteers will undergo clinical follow up for adverse events for 28 days following completion of the vaccination regimen. SAEs will be collected throughout the study. The duration of follow up reflects the desire to obtain safety data with the first use of MVA-NP+M1 manufactured on the AGE1.CR.pIX avian cell line.

5.2 Secondary Objectives

To assess the immunogenicity (cellular) of the candidate vaccine MVA-NP+M1 manufactured on the AGE1.CR.pIX avian cell line.

5.2.1 Secondary Outcome Measures

Immunogenicity will be assessed by a variety of immunological assays. Measures of immunogenicity may include:

- ELISpot to enumerate IFN-γ producing T cells
- the breadth of influenza-specific T-cells

Other exploratory immunology may be carried out in collaboration with other specialist laboratories. This would involve transfer of serum/plasma and/or peripheral blood mononuclear cells (PBMC), but samples would be anonymised. Volunteers will be consented for this.

6. INVESTIGATIONAL PRODUCT

MVA-NP+M1. The dose of MVA-NP+M1 to be used in this study will be 1.5 $x10^8$ pfu (converted from TCID50).

6.1 Manufacturing and presentation

The vaccine is supplied as a liquid in glass vials for intramuscular administration Each vial of MVA-NP+M1 contains a nominal 700 microliters volume in 10% sucrose, 0.1% pluronic acid, 25 mM Tris pH 8.0 buffer to a target final concentration of 1.2 x 10^9 TCID50/mL.

6.2 Supply

MVA-NP+M1 is manufactured under Good Manufacturing Practice (GMP) conditions by Emergent Biosolutions, USA on the cell line AGE1.CR.pIX. Final certification by a Qualified Person (QP) and associated labelling to trial will occur at the Clinical Biomanufacturing Facility (CBF) at the University of Oxford.

6.3 Storage

The vaccine is stored at nominal -80°C in a locked freezer, at the clinical site. All movement of the study vaccines between Emergent Biosolutions and the University of Oxford and between the locked freezer and clinic rooms will be documented in accordance with existing SOPs. Vaccine accountability, storage, shipment and handling will be in accordance with relevant local SOPs and forms.

6.4 Administration

On vaccination day, MVA-NP+M1 will be allowed to thaw to room temperature and administered within 1 hour. Vaccination will be performed and the IMPs handled according to the relevant SOPs. The vaccine will be administered intramuscularly into the deltoid of the non-dominant arm (preferentially). All volunteers will be observed in the unit for 1 hour (±10 minutes) after vaccination. During administration of the investigational products, Advanced Life Support drugs and resuscitation equipment will be immediately available for the management of anaphylaxis.

6.5 Rationale for selected vaccine dose

MVA-NP+M1, manufactured on the novel avian cell line, will be administered at 1.5 $x10^8$ pfu (converted from $TCID_{50}$). This dose has been chosen based on previous phase I and IIa

clinical trials of the MVA-NP+M1, manufactured on CEF, which have shown the vaccine is safe and immunogenic at the selected dose (FLU001, FLU002, FLU003, FLU004 and FLU005). Volunteers up to the age of 85 years received the proposed 1.5 x 10^8 pfu dose in FLU001 which was less reactogenic and better tolerated than the previously 2.5 x 10^8 pfu dose used in volunteers aged 18-50. The 1.5 x 10^8 pfu was chosen balancing reactogenicity and immunogenicity.

6.6 Minimising environmental contamination with genetically modified organisms (GMO)

The study will be performed in accordance with UK Genetically Modified Organisms (Contained Use) Regulations (2014). In order to minimise dissemination of the recombinant vectored vaccine virus into the environment, inoculation sites will be covered with a dressing after immunisation. This should absorb any virus that may leak out through the needle track. The dressing will be removed from the injection site after 30 minutes (+15/- 5 minutes) and will be disposed as GMO waste by autoclaving.

7 RECRUITMENT AND WITHDRAWAL OF TRIAL VOLUNTEERS

7.1 Identification of trial volunteers

Volunteers may be recruited by use of an advertisement +/- registration form formally approved by the ethics committee(s) and distributed or posted in the following places:

- In public places, including buses and trains, with the agreement of the owner/proprietor.
- In newspapers or other literature for circulation.
- On radio via announcements.
- On a website or social media site operated by our group or with the agreement of the owner or operator (including on-line recruitment through our web-site).
- By e-mail distribution to a group or list only with the express agreement of the network administrator or with equivalent authorisation.
- By email distribution to individuals who have already expressed an interest in taking part in any clinical trial at the Oxford Vaccine Centre.
- On stalls or stands at exhibitions or fairs.
- Via presentations (e.g. presentations at lectures or invited seminars).
- Direct mail-out: This will involve obtaining names and addresses of adults via the most recent Electoral Roll. The contact details of individuals who have indicated that they do not wish to receive postal mail-shots would be removed prior to the investigators being given this information. The company providing this service is registered under the Data Protection Act 1998. Investigators would not be given dates of birth or ages of individuals but the list supplied would only contain names of potentially eligible volunteers (as per the inclusion criteria).
- Oxford Vaccine Centre databases: We may contact individuals from databases of groups within the CCVTM (including the Oxford Vaccine Centre database) of previous trial participants who have expressed an interest in receiving information about all future studies for which they may be eligible.

7.2 Informed consent

All volunteers will sign and date the informed consent form before any study specific procedures are performed. The information sheet will be made available to the volunteer at least 24 hours prior to the screening visit. At the screening visit, the volunteer will be fully informed of all aspects of the trial, the potential risks and their obligations. The following general principles will be emphasised:

- Participation in the study is entirely voluntary
- Refusal to participate involves no penalty or loss of medical benefits

- The volunteer may withdraw from the study at any time
- The volunteer is free to ask questions at any time to allow him or her to understand the purpose of the study and the procedures involved
- The study involves research of an investigational vaccine
- There is no direct benefit from participating
- The volunteer's GP will be contacted to corroborate their medical history
- The volunteer's blood samples taken as part of the study will be stored indefinitely and samples may be sent outside of the UK and Europe to laboratories in collaboration with the University of Oxford. These will be anonymised.

The aims of the study and all tests to be carried out will be explained. The volunteer will be given the opportunity to ask about details of the trial, and will then have time to consider whether or not to participate. If they do decide to participate, they will sign and date two copies of the consent form, one for them to take away and keep, and one to be stored in the case report form (CRF) – this is a paper or electronic document used to collect data relating to a particular volunteer. These forms will also be signed and dated by the Investigator.

7.3 Inclusion and exclusion criteria

This study will be conducted in healthy adults, who meet the following inclusion and exclusion criteria

7.3.1 Inclusion Criteria

The volunteer must satisfy all the following criteria to be eligible for the study:

1. Healthy adults aged 18-50
2. Able and willing (in the Investigator's opinion) to comply with all study requirements
3. Willing to allow the investigators to discuss the volunteer's medical history with their General Practitioner
4. For females only, willingness to practice continuous effective contraception (see below) during the study and a negative pregnancy test on the day(s) of screening and vaccination (for women of child bearing potential only)
5. Agreement to refrain from blood donation during the course of the study
6. Provide written informed consent

7.3.2 Exclusion Criteria

The volunteer may not enter the study if any of the following apply:

1. Participation in another research study involving receipt of an investigational product in the 30 days preceding enrolment, or planned use during the study period
2. Prior receipt of an investigational or licensed vaccine likely to impact on interpretation of the trial data.
3. Administration of immunoglobulins and/or any blood products within the three months preceding the planned administration of the vaccine candidate
4. Any confirmed or suspected immunosuppressive or immunodeficient state, including HIV infection; asplenia; recurrent, severe infections and chronic (more than 14 days) immunosuppressant medication within the past 6 months (inhaled and topical steroids are allowed)
5. History of allergic disease or reactions likely to be exacerbated by any component of the vaccine
6. Any history of anaphylaxis in relation to vaccination
7. Pregnancy, lactation or willingness/intention to become pregnant during the study (for women of child bearing potential only)
8. History of cancer (except basal cell carcinoma of the skin and cervical carcinoma in situ)
9. History of serious psychiatric condition likely to affect participation in the study
10. Bleeding disorder (eg. Factor deficiency, coagulopathy or platelet disorder), or prior history of significant bleeding or bruising following IM injections or venepuncture
11. Any other serious chronic illness requiring hospital specialist supervision
12. Suspected or known current alcohol abuse as defined by an alcohol intake of greater than 42 units every week
13. Suspected or known injecting drug abuse in the 5 years preceding enrolment
14. Seropositive for hepatitis B surface antigen (HBsAg)
15. Seropositive for hepatitis C virus (antibodies to HCV)
16. Any clinically significant abnormal finding on screening biochemistry and haematology blood tests or urinalysis
17. Any other significant disease, disorder or finding which may significantly increase the risk to the volunteer because of participation in the study, affect the ability of the volunteer to participate in the study or impair interpretation of the study data

18. Inability of the study team to contact the volunteer's GP to confirm medical history and safety to participate

7.3.3 Effective contraception for female volunteers

Female volunteers are required to use an effective form of contraception during the course of the study (i.e. until their final follow up visit). Male subjects with female partners of child-bearing potential are not required to use barrier contraception whilst taking part in this study as the risk of excretion of the vaccine is negligible.

Acceptable forms of contraception for female volunteers include:

- Established use of oral, injected or implanted hormonal methods of contraception.
- Placement of an intrauterine device (IUD) or intrauterine system (IUS).
- Total abdominal hysterectomy
- Barrier methods of contraception (condom or occlusive cap with spermicide)
- Male sterilisation, if the vasectomised partner is the sole partner for the subject.
- True abstinence: when this is in line with the preferred and usual lifestyle of the subject.

  Periodic abstinence (e.g., calendar, ovulation, symptothermal, post-ovulation methods), declaration of abstinence for the duration of exposure to IMP, and withdrawal are not acceptable methods of contraception.

7.3.4 Prevention of 'Over Volunteering'

Volunteers will be excluded from the study if they are concurrently involved in another trial. In order to check this, volunteers will be asked to provide their National Insurance or Passport number (if they are not entitled to a NI number) and will be registered on a national database of participants in clinical trials (*www.tops.org.uk*).

7.3.5 Criteria for postponement of vaccination

The following events constitute contraindications to administration of vaccine at that point in time; if any one of these events occurs at the time scheduled for vaccination, the subject may be vaccinated at a later date, or withdrawn at the discretion of the Investigator.

- Acute disease at the time of vaccination (acute disease is defined as the presence of a moderate or severe illness with or without fever). All vaccines can be administered to persons with a minor illness such as diarrhoea, mild upper respiratory infection with or without low-grade febrile illness, i.e. temperature of ≤37.5°C/99.5°F.

- Temperature of >37.5°C (99.5°F) at the time of vaccination.

7.3.6 Withdrawal of Volunteers

In accordance with the principles of the current revision of the Declaration of Helsinki and any other applicable regulations, a volunteer has the right to withdraw from the study at any time and for any reason, and is not obliged to give his or her reasons for doing so. The Investigator may withdraw the volunteer at any time in the interests of the volunteer's health and well-being. In addition, the volunteer may withdraw/be withdrawn for any of the following reasons:

- Administrative decision by the Investigator.
- Ineligibility (either arising during the study or retrospectively, having been overlooked at screening).
- Significant protocol deviation.
- Volunteer non-compliance with study requirements.
- An AE, which requires discontinuation of the study involvement or results in inability to continue to comply with study procedures.

The reason for withdrawal will be recorded in the CRF. If withdrawal is due to an AE, appropriate follow-up visits or medical care will be arranged, with the agreement of the volunteer, until the AE has resolved, stabilised or a non-trial related causality has been assigned. Any volunteer who is withdrawn from the study may be replaced, if that is possible within the specified time frame. The chair of the LSC may recommend withdrawal of volunteers. Any volunteer who fails to attend for two or more follow-up visits during the study will be deemed to have withdrawn from the study.

If a volunteer withdraws from the study, blood samples collected before their withdrawal from the trial will be used/stored unless the volunteer specifically requests otherwise.

In all cases of subject withdrawal, excepting those of complete consent withdrawal, long-term safety data collection, including some procedures such as safety bloods, will continue as appropriate if subjects have received one or more vaccine doses.

7.4 Compliance with Dosing Regime

All vaccinations will be administered by the Investigator and recorded in the CRF. The study medication will be at no time in the possession of the volunteer and compliance will not, therefore, be an issue.

7.5 Pregnancy

Should a volunteer become pregnant during the trial, she will be followed up as other volunteers and in addition will be followed until pregnancy outcome. We will not routinely perform venepuncture in a pregnant volunteer.

8 TREATMENT OF TRIAL VOLUNTEERS

This section describes the clinical procedures for evaluating study participants and follow-up after administration of study vaccine.

8.1 Study procedures

All volunteers will have the same schedule of clinic attendances and procedures as indicated in the schedules of attendance (Table 4). The total volume of blood donated during the study will be approximately 180mL. Additional visits or procedures may be performed at the discretion of the investigators (e.g., further medical history and physical examination, urine microscopy in the event of positive urinalysis or additional blood tests when clinically relevant).

8.2 Observations

Pulse, blood pressure and temperature will be measured at the time-points indicated in the schedule of procedures and may also be measured as part of a physical examination if indicated at other time-points.

8.3 Blood tests and urinalysis

Blood will be drawn for the following laboratory tests and processed:

1. At Oxford University Hospitals' NHS Trust using NHS standard procedures:

- **Haematology;** Full Blood Count
- **Biochemistry;** Sodium, Potassium, Urea, Creatinine, Albumin, Liver Function Tests (ALT, ALP and bilirubin)
- **Diagnostic serology;** HBsAg, HCV antibodies, HIV antibodies (specific consent will be gained prior to testing blood for these blood-borne viruses)

Additional safety blood tests may be performed if clinically relevant at the discretion of the medically qualified investigators. These generally include, but are not limited to, AST, GGT and a coagulation screen.

2. At University of Oxford research laboratories:

- **Exploratory Immunology**; Immunogenicity will be assessed by a variety of immunological assays. This may include *ex vivo* ELISpot assays for interferon gamma and flow cytometry assay. Other exploratory immunological assays including cytokine analysis, DNA analysis, assessments of humoral immunity and gene

expression studies amongst others may be performed at the discretion of the Investigators.

3. **Urinalysis;** Urine will be tested for protein, blood and glucose at screening. For female volunteers only, urine will be tested for beta-human chorionic gonadotrophin (β-HCG) at screening and immediately prior to vaccination. Additional urine tests may be performed if clinically relevant at the discretion of the medically qualified investigators. These generally include, but are not limited to albumin:creatinine ratio and culture .

4. Collaboration with other specialist laboratories in the UK, Europe and outside of Europe for further exploratory tests may occur. This would involve the transfer of serum, urine or plasma and/or PBMC to these laboratories, but these would remain anonymised. Informed consent for this will be gained from volunteers. Immunological assays will be conducted according to local SOPs.

Subjects will be informed that there may be leftover samples of their blood (after all testing for this study is completed), and that such samples may be stored indefinitely in the Oxford Vaccine Centre Biobank for possible future research (exploratory immunology), including human DNA and RNA analyses to search for correlates of vaccine immunogenicity and efficacy. Blood samples will be stored in compliance with the Human Tissue Act. Subjects will be able to decide if they will permit such future use of any leftover samples. With the volunteers' informed consent, any leftover cells, urine and serum/plasma will be frozen indefinitely for future analysis of influenza-specific or vaccine-related responses. If a subject elects not to permit this, all of that subject's leftover samples will be discarded after the required period of storage to meet Good Clinical Practice (GCP) and regulatory requirements.

8.4 Study visits

The study visits and procedures will be undertaken by one of the clinical trials team members. The procedures to be included in each visit are documented in the schedule of attendances (Table 4). Each visit is assigned a time-point and a window period, within which the visit will be conducted.

8.4.1 Screening visit

All potential volunteers will have a screening visit, which may take place up to 90 days prior to vaccination. Informed consent will be taken before screening, as described in section 7.2.

If consent is obtained, the procedures indicated in the schedule of attendances will be undertaken including a medical history, physical examination and blood tests. To avoid unnecessary additional venepuncture, if the appropriate blood test results for screening are available for the same volunteer from a screening visit for another Jenner Institute Clinical Trials group vaccine study, these results may be used for assessing eligibility (provided the results date is within the 3 months preceding enrolment in FLU008).

The subject's general practitioner will be contacted with the written permission of the subject after screening to ascertain any significant medical history and as notification that the subject has volunteered for the study. During the screening the volunteers will be asked to provide their National Insurance or passport number so that this can be entered on to a national database which helps prevent volunteers from participating in more than one clinical trial simultaneously or over-volunteering for clinical trials (www.tops.org.uk). Abnormal clinical findings from the urinalysis or blood tests at screening will be assessed by the lead clinician according to the relevant SOP. Abnormal blood tests following screening will be assessed according to site-specific laboratory adverse event grading tables which are filed in the trial master file (TMF) or the Investigator Site File (ISF). Any abnormal test result deemed clinically significant may be repeated to ensure it is not a single occurrence. If an abnormal finding is deemed to be clinically significant, the volunteer will be informed and appropriate medical care arranged with the permission of the volunteer.

The eligibility of the volunteer will be reviewed at the end of the screening visit and again when all results from the screening visit have been considered. Decisions to exclude the volunteer from enrolling in the trial or to withdraw a volunteer from the trial will be at the discretion of the Investigator. If eligible, a day 0 visit will be scheduled for the volunteer to receive the vaccine.

8.4.2 Day 0: Enrolment and vaccination visit

Volunteers will not be considered enrolled in the study until they have received a vaccine. Before vaccination, the eligibility of the volunteer will be reviewed. Pulse, blood pressure and temperature will be observed and if necessary, a medical history and physical examination may be undertaken to determine need to postpone vaccination depending on criteria listed in section 7.3.5. Vaccinations will be administered as described below.

8.4.2.1 Vaccination

Before each vaccination, the on-going eligibility of the volunteer will be reviewed. The vaccines will be administered intramuscularly into the deltoid of the non-dominant arm (preferentially), according to the relevant SOP. The injection site will be covered with a sterile dressing and the volunteer will stay in the CCVTM for 60 minutes (± 10 minutes) after

vaccination, in case of immediate adverse events. Observations will be taken 30 minutes (± 10 minutes) after vaccination and the sterile dressing removed and injection site inspected.

After vaccination the following procedures will be undertaken:

Volunteers will be given an oral thermometer, tape measure and diary card (paper or electronic), with instructions on use, along with the emergency 24 hour telephone number to contact the on-call study physician if needed. Volunteers will be instructed on how to self-assess the severity of these AEs. There will also be space on the diary card to self-document unsolicited AEs, and whether medication was taken to relieve the symptoms. Diary cards will collect information on the timing and severity of the following solicited AEs:

Table 3. Solicited AEs as collected on post vaccination diary cards

Local solicited AEs	Systemic solicited AEs
Pain	Fever
Redness	Feverishness/Chills
Warmth	Joint pains
Itch	Muscle pains
	Fatigue
	Headache
	Malaise
	Nausea

8.4.2.2 Sequence of Enrolment and Vaccination of Volunteers

The first volunteer will be vaccinated alone and the profile of adverse events assessed. No other volunteers will be vaccinated until at least 48 hours have elapsed following the first volunteer being vaccinated. The CI and the LSM will be asked to provide the decision on whether to proceed after the review of the first volunteer. Provided no serious adverse reactions have occurred then a further two volunteers will be vaccinated at least 1 hour apart from each other. An independent safety review will be carried out by the LSM after the first 3 volunteers in the group have been vaccinated. This review will include the results of safety blood tests at day 7 post vaccination. The CI and the LSM will be asked to provide the decision on whether to proceed with vaccination of the remaining 3 volunteers.

8.4.3 Subsequent visits: days 2, 7, 21 and 28.

Follow-up visits will take place 48 hours (±24h), 7 days (± 2 days), 21 days (± 2 days) and 28 days (± 2 days) after vaccination. Volunteers will be assessed for local and systemic adverse events, interim history, physical examination, review of diary cards (paper or electronic) and

blood tests at these time points as detailed in the schedule of attendances. Blood will also be taken for exploratory immunology.

If volunteers experience adverse events (laboratory or clinical), which the investigator (physician), CI and/or LSM determine necessary for further close observation, the volunteer may be admitted to an NHS hospital for observation and further medical management under the care of the Consultant on call.

Table 4. Schedule of visits for volunteers

**Attendance number**	**1[S]**	**2**	**3**	**4**	**5**	**6**
Timeline (days)	<90	0	2	7	21	28
(weeks)		0		1	3	4
Time window (days)			±1	±2	±2	±2
Informed consent	X					
Review contraindications, inclusion and exclusion criteria	X	X				
Medical history, physical examination	X	(X)	(X)	(X)	(X)	(X)
Vital signs^	X	X	X	X	X	X
Vaccination		X				
Diary card provided		X				
Diary card collected						X
Record adverse events			X	X	X	X
Biochemistry[$] / Haematology (mL)	5	5	5	5		5
Urinalysis (protein, glucose, blood)	X					
Urinary b-HCG (women only)	X	X				
HBsAg, HCV Ab, HIV serology (mL)	5					
Exploratory immunology[£]		50		50	50	
**Blood volume per visit**	**10**	**55**	**5**	**55**	**50**	**5**
**Cumulative blood volume****	**10**	**65**	**70**	**125**	**175**	**180**

[S] *= screening visit; (X) = if considered necessary ^ = Vital signs includes pulse, blood pressure and temperature;* [$] *= Biochemistry will include Sodium, Potassium, Urea, Creatinine, Albumin and Liver function tests.* [£] = Exploratory Immunology will include IFN-γ T cell ELISPOT and further experimental assays to be confirmed at a later date. *** Cumulative blood volume for volunteers if bloods taken as per schedule, and excluding any repeat safety blood test that may be necessary.*

9 ASSESSMENT OF SAFETY

Safety will be assessed by the frequency, incidence and nature of adverse events and serious adverse events arising during the study.

9.1 Interim Safety Reviews

Interim safety reviews with the Chair of the LSC (LSM) are planned after the first three volunteers, prior to vaccination of the remaining recruited volunteers. Safety reviews consist of an assessment of the profile and severity of adverse events reported and will include the results of safety blood tests at day 7 post vaccination. Interim safety data may also be made available to manufacturers (in coded format) as specified in the contract with the manufacturer(s).

9.2 Definitions

9.2.1 Adverse Event (AE)

An AE is any untoward medical occurrence in a volunteer, which may occur during or after administration of an Investigational Medicinal Product (IMP) and does not necessarily have a causal relationship with the intervention. An AE can therefore be any unfavourable and unintended sign (including an abnormal laboratory finding), symptom or disease temporally associated with the study intervention, whether or not considered related to the study intervention.

9.2.2 Adverse Reaction (AR)

An AR is any untoward or unintended response to an IMP. This means that a causal relationship between the IMP and an AE is at least a reasonable possibility, i.e., the relationship cannot be ruled out. All cases judged by the reporting medical Investigator as having a reasonable suspected causal relationship to an IMP (i.e. possibly, probably or definitely related to an IMP) will qualify as adverse reactions.

9.2.3 Unexpected Adverse Reaction

An adverse reaction, the nature or severity of which is not consistent with the applicable product information (e.g. IB for an unapproved IMP).

9.2.4 Serious Adverse Event (SAE)

A SAE is an AE that results in any of the following outcomes, whether or not considered related to the study intervention.

- Death
- Life-threatening event (i.e., the volunteer was, in the view of the Investigator, at immediate risk of death from the event that occurred). This does not include an AE that, if it occurred in a more severe form, might have caused death.
- Persistent or significant disability or incapacity (i.e., substantial disruption of one's ability to carry out normal life functions).
- Hospitalisation, regardless of length of stay, even if it is a precautionary measure for continued observation. Hospitalisation (including inpatient or outpatient hospitalisation for an elective procedure) for a pre-existing condition that has not worsened unexpectedly does not constitute a serious AE.
- An important medical event (that may not cause death, be life threatening, or require hospitalisation) that may, based upon appropriate medical judgment, jeopardise the volunteer and/or require medical or surgical intervention to prevent one of the outcomes listed above. Examples of such medical events include allergic reaction requiring intensive treatment in an emergency room or clinic, blood dyscrasias, or convulsions that do not result in inpatient hospitalisation.
- Congenital anomaly or birth defect.

9.2.5 Serious Adverse Reaction (SAR)

An adverse event (expected or unexpected) that is both serious and, in the opinion of the reporting Investigator or Sponsors, believed to be possibly, probably or definitely due to an IMP or any other study treatments, based on the information provided.

9.2.6 Suspected Unexpected Serious Adverse Reaction (SUSAR)

A serious adverse reaction, the nature and severity of which is not consistent with the information about the medicinal product in question set out in the IB.

9.3 Foreseeable Adverse Reactions:

The foreseeable ARs following vaccination with MVA NP+M1 include injection site pain, erythema, warmth, swelling, pruritus, myalgia, arthralgia, headache, fatigue, fever, feverishness, malaise, nausea, muscular and articular pain.

9.4 Expected Serious Adverse Events

No serious adverse events are expected in this study.

9.5 Causality Assessment

For every AE, an assessment of the relationship of the event to the administration of the vaccine will be undertaken by the CI-delegated clinician. An intervention-related AE refers to an AE for which there is a probable or definite relationship to administration of a vaccine. An interpretation of the causal relationship of the intervention to the AE in question will be made, based on the type of event; the relationship of the event to the time of vaccine administration; and the known biology of the vaccine therapy (Table 5). Alternative causes of the AE, such as the natural history of pre-existing medical conditions, concomitant therapy, other risk factors and the temporal relationship of the event to vaccination will be considered and investigated. Causality assessment will take place during planned safety reviews, interim analyses (e.g. if a holding or stopping rule is activated) and at the final safety analysis, except for SAEs, which should be assigned by the reporting investigator.

Table 5. Guidelines for assessing the relationship of vaccine administration to an AE.

0	**No Relationship**	No temporal relationship to study product ***and***<br>Alternate aetiology (clinical state, environmental or other interventions); ***and***<br>Does not follow known pattern of response to study product
1	**Unlikely**	Unlikely temporal relationship to study product ***and***<br>Alternate aetiology likely (clinical state, environmental or other interventions) ***and***<br>Does not follow known typical or plausible pattern of response to study product.
2	**Possible**	Reasonable temporal relationship to study product; ***or***<br>Event not readily produced by clinical state, environmental or other interventions; ***or***<br>Similar pattern of response to that seen with other vaccines
3	**Probable**	Reasonable temporal relationship to study product; ***and***<br>Event not readily produced by clinical state, environment, or other interventions ***or***<br>Known pattern of response seen with other vaccines
4	**Definite**	Reasonable temporal relationship to study product; ***and***<br>Event not readily produced by clinical state, environment, or other interventions; ***and***<br>Known pattern of response seen with other vaccines

9.6 Reporting Procedures for All Adverse Events

All local and systemic AEs occurring in the 28 days following each vaccination observed by the Investigator or reported by the volunteer, whether or not attributed to study medication, will be recorded. Recording and reporting of all AEs will take place as detailed in the relevant SOP. All AEs that result in a volunteer's withdrawal from the study will be followed up until a satisfactory resolution occurs, or until a non-study related causality is assigned (if the volunteer consents to this). Serious adverse events (SAEs) will be collected throughout the entire trial period.

9.6.1 Reporting Procedures for Serious AEs

In order to comply with current regulations on serious adverse event reporting to regulatory authorities, the event will be documented accurately and notification deadlines respected. SAEs will be reported on the SAE forms to members of the study team immediately the Investigators become aware of their occurrence, as described in relevant safety reporting SOPs. Copies of all reports will be forwarded for review to the Chief Investigator (as the Sponsor's representative) within 24 hours of the Investigator being aware of the suspected SAE. The local safety monitor (LSM) will be notified of SAEs that are deemed possibly, probably or definitely related to study interventions; the LSM will be notified immediately (within 24 hours) of the Investigators' being aware of their occurrence. SAEs will not normally be reported immediately to the ethical committee(s) unless there is a clinically important increase in occurrence rate, an unexpected outcome, or a new event that is likely to affect safety of trial volunteers, at the discretion of the Chief Investigator and/or LSM. In addition to the expedited reporting above, the Investigator shall include all SAEs in the annual Development Safety Update Report (DSUR) report.

9.6.2 Reporting Procedures for SUSARS

The Chief Investigator will report all SUSARs to the MHRA and ethical committee(s) within required timelines (15 days for all SUSARs, unless life threatening in which case 7 days, with a final report within a further 8 days (total 15). The Chief Investigator will also inform all Investigators concerned of relevant information about SUSARs that could adversely affect the safety of participants. All SUSARs and deaths occurring during the study will be reported to the Sponsor. For all deaths, available autopsy reports and relevant medical reports will be made available for reporting to the relevant authorities.

9.6.3 Development Safety Update Report

A Development Safety Update Report (DSUR) will be submitted by the Sponsor to the competent authority and ethical committee on the anniversary of the first approval date from the regulatory authority for each IMP.

9.7 Assessment of severity

The severity of clinical and laboratory adverse events will be assessed according to the scales in Table 6 -Table 8.

Table 6. Severity grading criteria for local adverse events

Adverse Event	Grade	Intensity
Erythema at injection site*	1	>3 - ≤50 mm
	2	>50 - ≤100 mm
	3	>100 mm
Swelling at injection site	1	>3 - ≤20 mm
	2	>20 - ≤50 mm
	3	>50 mm
Ulceration/necrosis of skin at injection site	1	-
	2	-
	3	Any

*erythema or swelling ≤3mm is an expected consequence of skin puncture and will therefore not be considered an adverse event.

Table 7. Severity grading criteria for physical observations.

	Grade 1 (mild)	Grade 2 (moderate)	Grade 3 (severe)
Fever (oral)	37.6°C - 38.0°C	38.1°C – 39.0°C	>39.0°C
Tachycardia (bpm)*	101 - 115	116 – 130	>130
Bradycardia (bpm)**	50 – 54	40 – 49	<40
Systolic hypertension (mmHg)	141 - 159	160 – 179	≥180
Systolic hypotension (mmHg)***	85 - 89	80 – 84	<80
Diastolic hypertension (mmHg)	91 - 99	100 – 109	≥110

*Taken after ≥10 minutes at rest
**Use clinical judgement when characterising bradycardia among some healthy subject populations, for example, conditioned athletes.
***Only if symptomatic (e.g. dizzy/ light-headed)

Table 8. Severity grading criteria for local and systemic AEs.

**GRADE 0**	None: Symptom not experienced
**GRADE 1**	Mild: Short-lived or mild symptoms; medication may be required. No limitation to usual activity
**GRADE 2**	Moderate: Mild to moderate limitation in usual activity. Medication may be required.
**GRADE 3**	Severe: Considerable limitation in activity. Medication or medical attention required.

9.8 Procedures to be followed in the event of abnormal findings

Laboratory parameters for inclusion/exclusion in the trial will be considered on an individual basis, with investigator discretion for interpretation of results and the need for repeated tests. In general, volunteers will be excluded if a result at screening constitutes what would qualify as a grade 1 (or higher) laboratory adverse event, according to the site-specific laboratory adverse event tables (stored in TMF or ISF). Abnormal clinical findings from medical history, examination or blood tests will be assessed as to their clinical significance throughout the trial. If a test is deemed clinically significant, it may be repeated, to ensure it is not a single occurrence. If a test remains clinically significant, the volunteer will be informed and appropriate medical care arranged as appropriate and with the permission of the volunteer. Decisions to exclude the volunteer from enrolling in the trial or to withdraw a volunteer from the trial will be at the discretion of the Investigator.

9.9 Local Safety Committee

A Local Safety Committee (LSC) will be appointed to provide real-time safety oversight. The LSC will review SAEs deemed possibly, probably or definitely related to study interventions. The LSC will be notified within 24 hours of the Investigators' being aware of their occurrence. The LSC has the power to place the study on hold if deemed necessary following a study intervention-related SAE. At the time of writing the LSC will be chaired by Dr Brian Angus, a Clinical Tutor in Medicine, Honorary Consultant Physician and Director, Centre for Tropical Medicine at the University of Oxford. There will be a minimum of two other appropriately qualified committee members. All correspondence between Investigator and LSC will be conveyed by the Investigator to the trial Sponsor.

The chair of the LSC may be contacted for advice and independent review by the Investigator or trial Sponsor in the following situations:

- Following any SAE deemed to be possibly, probably, or definitely related to a study intervention.

- Any other situation where the Investigator or trial Sponsor feels independent advice or review is important.

9.9.1 Safety Profile Review

The safety profile will be assessed on an on-going basis by the Investigators. The LSC will perform independent external safety reviews after vaccination of the first 3 volunteers. The Chief investigator and relevant Investigators (as per the trial delegation log) will also review safety issues and SAEs as they arise.

9.10 Safety Holding Rules

Safety holding rules have been developed considering the fact this is the first time the MVA-NP+M1 vaccine, developed on the novel immortalised duck retinal cell line AGE1.CR.pIX, is used. 'Solicited adverse events' are those listed as foreseeable adverse events in section 9 of the protocol, occurring within the first 7 days after vaccination (day of vaccination and six subsequent days). 'Unsolicited adverse events' are adverse events other than the foreseeable AEs occurring within the first 7 days or any AEs occurring after 7 days post vaccination.

The group holding rules are as follows:

- **Solicited local adverse events:** If 2 or more volunteers present the same Grade 3 solicited local adverse event beginning within 2 days after vaccination (day of vaccination and one subsequent day) and persisting at Grade 3 for >48 hrs

- **Solicited systemic adverse events:** If 2 or more volunteers present the same Grade 3 solicited systemic adverse event beginning within 2 days after vaccination (day of vaccination and one subsequent day) and persisting at Grade 3 for >48hrs.

- **Unsolicited adverse events:** If 2 or more volunteers develop the same Grade 3 unsolicited adverse event (including the same laboratory adverse event) that is considered possibly, probably or definitely related to vaccination and persists at Grade 3 for >48hrs.

- **A life-threatening reaction considered possibly, probably or definitely related to vaccination occurs**

- **A serious adverse event considered possibly, probably or definitely related to vaccination occurs**

- **Death considered possibly, probably or definitely related to vaccination**

If a volunteer has an acute illness (moderate or severe illness with or without fever) or a fever (oral temperature greater than 37.5°C) at the scheduled time of administration of investigational product, the volunteer will not receive the vaccine at that time. The vaccine may be administered to that volunteer at a later date within the time window specified in the protocol (see Table 4) or they may be withdrawn from the study at the discretion of the Investigator.

If a holding rule has been met and following an internal safety review it is deemed appropriate to restart dosing, a request to restart dosing with pertinent data must be submitted to the regulatory authority as a request for a substantial amendment. The internal safety review will consider:

- The relationship of the AE or SAE to the vaccine.
- The relationship of the AE or SAE to the vaccine dose, or other possible causes of the event.
- If appropriate, additional screening or laboratory testing for other volunteers to identify those who may develop similar symptoms and alterations to the current Participant Information Sheet (PIS) are discussed.
- New, relevant safety information from ongoing research programs on the various components of the vaccine.

The local ethics committee and vaccine manufacturers will also be notified if a holding rule is activated or released.

All vaccinated volunteers will be followed for safety reasons until resolution or stabilization (if determined to be chronic sequelae) of their AEs.

In addition to the pre-defined criteria, the study can be put on hold upon advice of the Local Safety Monitor, Chief Investigator, Study Sponsor, regulatory authority, Ethical Committee(s) or Local Safety Committee, for any single event or combination of multiple events which, in their professional opinion, jeopardise the safety of the volunteers or the reliability of the data.

10 STATISTICS

Sample Size Selection and Statistical analysis

This is a descriptive safety study, where volunteers will be vaccinated with MVA-NP+M1 alone. A maximum of 6 volunteers will be vaccinated in total. Safety data will be presented according to frequency, severity and duration of adverse events. This sample size should

allow an initial estimation to be made of the frequency and magnitude of outcome measures.

Non-parametric tests will be used to determine differences in the primary immunogenicity outcome (ELISpot) data, testing for differences in responses between time points within a group.

11. QUALITY CONTROL AND QUALITY ASSURANCE PROCEDURES

11.1 Investigator procedures

Approved site-specific standard operating procedures (SOPs) will be used at clinical and laboratory sites.

11.2 Monitoring

Monitoring will be performed independently, according to ICH GCP. The monitors will verify that the clinical trial is conducted and data are generated, documented and reported in compliance with the protocol, GCP and the applicable regulatory requirements. The site will provide direct access to all trial related source data/documents and reports for the purpose of monitoring and auditing by the Sponsor and inspection by local and regulatory authorities.

11.3 Modification to protocol

No substantial amendments to this protocol will be made without consultation with, and agreement of the Sponsor. Any substantial amendments to the trial that appear necessary during the course of the trial must be discussed by the Investigator and Sponsor concurrently. If agreement is reached concerning the need for an amendment, it will be produced in writing by the Chief Investigator and will be made a formal part of the protocol following ethical and regulatory approval.

The Investigator is responsible for ensuring that changes to an approved trial, during the period for which regulatory and ethical committee(s) approval has already been given, are not initiated without regulatory and ethical committee(s)' review and approval except to eliminate apparent immediate hazards to the subject.

11.4 Protocol deviation

Any deviations from the protocol will be documented in a protocol deviation form and filed in the trial master file.

11.5 Audit & inspection

The QA manager may perform internal audits to check that the trial is being conducted; data recorded, analysed and accurately reported according to the protocol, Sponsor's SOPs and in compliance with ICH GCP. The audits will also include laboratory activities according to an

agreed audit schedule. The internal audits will supplement the external monitoring process and will review processes not covered by the external monitor.

The Sponsor, trial sites, and ethical committee(s) may carry out audit to ensure compliance with the protocol, GCP and appropriate regulations. GCP inspections may also be undertaken by the MHRA to ensure compliance with protocol and the Medicines for Human Use (Clinical Trials) Regulations 2004. The Sponsor will assist in any inspections and will formally respond to the MHRA as part of the inspection procedure.

11.6 Serious Breaches

The Medicines for Human Use (Clinical Trials) Regulations contain a requirement for the notification of "serious breaches" to the MHRA within 7 days of the Sponsor becoming aware of the breach.

A serious breach is defined as "A breach of GCP or the trial protocol which is likely to effect to a significant degree

(a) the safety or physical or mental integrity of the subjects of the trial; or

(b) the scientific value of the trial".

In the event that a serious breach is suspected the Sponsor will be informed within one working day.

11.7 Trial Progress

The progress of the trial will be overseen by the Chief Investigator. Trial updates will be provided at regular study team meetings.

11.8 Publication Policy

Publication by the site of any data from this study must be carried out in accordance with the clinical trial agreement and with prior permission from the Sponsor. The Investigator may prepare data derived from the study for publication. Such data will be submitted to the Sponsor for review and comment prior to publication. In order to ensure that the Sponsor will be able to make comments and suggestions, material for public dissemination will be submitted to the Sponsor for review at least thirty (30) days prior to submission for publication, public dissemination, or review by a publication committee. The Investigator agrees that all reasonable comments made by the Sponsor in relation to a proposed publication will be incorporated into the publication. The Sponsor will be entitled to delay the publication for a period of up to six (6) months from the date of first submission to the

Sponsor in order to enable the Sponsor to take steps to protect its proprietary information and Intellectual Property Rights and Know How.

The Sponsor may present at symposia, national or regional professional meetings, and publish in journals, thesis or dissertations, or otherwise of their own choosing, methods and results of the study and in particular, post a summary of study results in on-line clinical trials registers before or after publication by any other method. In the event the Sponsor coordinates a multi-centre publication, the participation of the Investigator shall be determined in accordance with the Sponsor's policy and generally accepted standards for authorship. If the Investigator is a named author of the multi-centre publication, the Investigator will have access to the study data from all Clinical Trial sites as necessary to participate fully in the development of the multi-centre publication.

Any publication based on data or other results of the study from individual study sites shall not be made before the first multi-centred publication or one year after completion of the study, whichever is the earlier.

12. ETHICS AND REGULATORY CONSIDERATIONS

12.1 Declaration of Helsinki

The Investigators will ensure that this study is conducted according to the principles of the current revision of the Declaration of Helsinki.

12.2 Guidelines for Good Clinical Practice

The Investigators will ensure that this study is conducted in full conformity with the Good Clinical Practice (GCP), the requirements of the Medicines for Human Use (Clinical Trial) Regulations 2004, and local regulatory requirements.

12.3 Informed Consent

Written, informed consent will be obtained, as described in section 7.2

12.4 Approvals

The protocol, informed consent form, participant information sheet and any proposed advertising material will be submitted to an appropriate Research Ethics Committee (REC), HRA (where required), regulatory authorities (MHRA in the UK), and host institution(s) for written approval.

The Investigator will submit and, where necessary, obtain approval from the above parties for all substantial amendments to the original approved documents.

12.5 Volunteer Confidentiality

All data will be anonymised: volunteer data will be identified by a unique study number in the CRF and database. A separate confidential file containing identifiable information will be stored in a secured location in accordance with the Data Protection Act 1998. Only the Sponsor representative, Investigators, the clinical monitor, the REC and the MHRA will have access to the records. Photographs taken of vaccination sites (if required, with the volunteer's written, informed consent) will not include the volunteer's face and will be identified by the date, trial code and subject's unique identifier. Once developed, photographs will be stored as confidential records, as above. This material may be shown to other professional staff, used for educational purposes, or included in a scientific publication.

13. DATA HANDLING AND RECORD KEEPING

13.1 Data Handling

The Chief Investigator will be responsible for all data that accrues from the study. The data will be entered into the volunteers' CRFs in a paper and/or electronic format (using OpenClinica™ database). Electronic data will be stored on secure servers which are outsourced by OpenClinica™. Data will be entered in a web browser on PCs in the CCVTM building and then transferred to the OpenClinica Database by encrypted (Https) transfer. OpenClinica™ meets FDA part 11B standards. This includes safety data, laboratory data (both clinical and immunological) and outcome data.

13.2 Record Keeping

The Investigators will maintain appropriate medical and research records for this trial, in compliance with ICH E6 GCP and regulatory and institutional requirements for the protection of confidentiality of volunteers. The Chief Investigator, co-Investigators and clinical research nurses will have access to records. The Investigators will permit authorised representatives of the Sponsor(s), as well as ethical and regulatory agencies to examine (and when required by applicable law, to copy) clinical records for the purposes of quality assurance reviews, audits and evaluation of the study safety and progress.

13.3 Source Data and Case Report Forms (CRFs)

All protocol-required information will be collected in CRFs designed by the Investigator. All source documents will be filed in the CRF. Source documents are original documents, data, and records from which the volunteer's CRF data are obtained. For this study, these will include, but are not limited to, volunteer consent form, blood results, GP response letters, laboratory records, diaries, and correspondence. In the majority of cases, CRF entries will be considered source data as the CRF is the site of the original recording (i.e. there is no other written or electronic record of data). In this study this will include, but is not limited to medical history, medication records, vital signs, physical examination records, urine assessments, blood results, adverse event data and details of vaccinations. All source data and volunteer CRFs will be stored securely.

13.4 Data Protection

The study protocol, documentation, data and all other information generated will be held in strict confidence. No information concerning the study or the data will be released to any unauthorised third party, without prior written approval of the sponsor.

14 FINANCING AND INSURANCE

14.2 Financing

The study is funded by Vaccitech.

14.3 Insurance

Vaccitech has a specialist insurance policy in place which would operate in the event of any participant suffering harm as a result of their involvement in the research.

14.4 Compensation

Volunteers will be compensated for their time and for the inconvenience caused by procedures. They will be compensated £25 for attending the screening visit.

For all other trial visits as outlined in Table 4 compensation will be calculated according to the following:

- Travel expenses: £10 per visit. Where travel expenses are greater than £10 per visit because the volunteer lives outside the city of the trial site, the volunteer will be given further reimbursement to meet the cost of travel necessary for study visits.

- Inconvenience of blood tests: £10 per blood donation

- Time required for visit: £20 per hour

References

1. Lagace-Wiens PR, Rubinstein E, Gumel A. Influenza epidemiology--past, present, and future. Critical care medicine. 2010;38(4 Suppl):e1-9.

2. Treanor J. Influenza Viruses, Including Avian Influenza and Swine Influenza. Principles and Practice of Infectious Diseases Mandell GL, Bennett JE, Dolin R (Eds).2:2060–85.

3. Who. Influenza fact sheet No. 211. 2014.

4. Molinari NA, Ortega-Sanchez IR, Messonnier ML, Thompson WW, Wortley PM, Weintraub E, et al. The annual impact of seasonal influenza in the US: measuring disease burden and costs. Vaccine. 2007;25(27):5086-96.

5. Dawood FS, Iuliano AD, Reed C, Meltzer MI, Shay DK, Cheng PY, et al. Estimated global mortality associated with the first 12 months of 2009 pandemic influenza A H1N1 virus circulation: a modelling study. Lancet Infect Dis. 2012;12(9):687-95.

6. Babakir-Mina M, Balestra E, Perno CF, Aquaro S. Influenza virus A (H5N1): a pandemic risk? The new microbiologica. 2007;30(2):65-78.

7. Kilbourne ED, Smith C, Brett I, Pokorny BA, Johansson B, Cox N. The total influenza vaccine failure of 1947 revisited: major intrasubtypic antigenic change can explain failure of vaccine in a post-World War II epidemic. Proceedings of the National Academy of Sciences of the United States of America. 2002;99(16):10748-52.

8. Bardenheier BH, Strikas R, Kempe A, Stokley S, Ellis J. Influenza vaccine supply, 2005-2006: did we come up short? BMC health services research. 2007;7:66.

9. Hannoun C, Megas F, Piercy J. Immunogenicity and protective efficacy of influenza vaccination. Virus research. 2004;103(1-2):133-8.

10. Hayward AC, Wang L, Goonetilleke N, Fragaszy EB, Bermingham A, Copas A, et al. Natural T Cell Mediated Protection Against Seasonal and Pandemic Influenza: Results of the Flu Watch Cohort Study. American Journal of Respiratory and Critical Care Medicine. 2015.

11. Sridhar S, Begom S, Bermingham A, Hoschler K, Adamson W, Carman W, et al. Cellular immune correlates of protection against symptomatic pandemic influenza. Nat Med. 2013;19(10):1305-12.

12. Berthoud TK, Hamill M, Lillie PJ, Hwenda L, Collins KA, Ewer KJ, et al. Potent CD8+ T-cell immunogenicity in humans of a novel heterosubtypic influenza A vaccine, MVA-NP+M1. Clin Infect Dis. 2011;52(1):1-7.

13. Antrobus RD, Lillie PJ, Berthoud TK, Spencer AJ, McLaren JE, Ladell K, et al. A T cell-inducing influenza vaccine for the elderly: safety and immunogenicity of MVA-NP+M1 in adults aged over 50 years. PLoS One. 2012;7(10):e48322.

14. Antrobus RD, Berthoud TK, Mullarkey CE, Hoschler K, Coughlan L, Zambon M, et al. Coadministration of seasonal influenza vaccine and MVA-NP+M1 simultaneously achieves potent humoral and cell-mediated responses. Mol Ther. 2014;22(1):233-8.

15. Lillie PJ, Berthoud TK, Powell TJ, Lambe T, Mullarkey C, Spencer AJ, et al. Preliminary assessment of the efficacy of a T-cell-based influenza vaccine, MVA-NP+M1, in humans. Clin Infect Dis. 2012;55(1):19-25.

16. Mayr A, Stickl H, Muller HK, Danner K, Singer H. [The smallpox vaccination strain MVA: marker, genetic structure, experience gained with the parenteral vaccination and behavior in organisms with a debilitated defence mechanism (author's transl)]. Zentralblatt fur Bakteriologie, Parasitenkunde, Infektionskrankheiten und Hygiene Erste Abteilung Originale Reihe B: Hygiene, Betriebshygiene, praventive Medizin. 1978;167(5-6):375-90.

17. Hochstein-Mintzel V, Hanichen T, Huber HC, Stickl H. [An attenuated strain of vaccinia virus (MVA). Successful intramuscular immunization against vaccinia and variola (author's transl)]. Zentralbl Bakteriol Orig A. 1975;230(3):283-97.

18. Goepfert PA, Elizaga ML, Sato A, Qin L, Cardinali M, Hay CM, et al. Phase 1 safety and immunogenicity testing of DNA and recombinant modified vaccinia Ankara vaccines expressing HIV-1 virus-like particles. The Journal of infectious diseases. 2011;203(5):610-9.

19. Howles S, Guimaraes-Walker A, Yang H, Hancock G, di Gleria K, Tarragona-Fiol T, et al. Vaccination with a modified vaccinia virus Ankara (MVA)-vectored HIV-1 immunogen induces modest vector-specific T cell responses in human subjects. Vaccine. 2010;28(45):7306-12.

20. Scriba TJ, Tameris M, Mansoor N, Smit E, van der Merwe L, Isaacs F, et al. Modified vaccinia Ankara-expressing Ag85A, a novel tuberculosis vaccine, is safe in adolescents and children, and induces polyfunctional CD4+ T cells. European journal of immunology. 2010;40(1):279-90.

21. Swadling L, Capone S, Antrobus RD, Brown A, Richardson R, Newell EW, et al. A human vaccine strategy based on chimpanzee adenoviral and MVA vectors that primes, boosts, and sustains functional HCV-specific T cell memory. Sci Transl Med. 2014;6(261):261ra153.

22. Dangoor A, Lorigan P, Keilholz U, Schadendorf D, Harris A, Ottensmeier C, et al. Clinical and immunological responses in metastatic melanoma patients vaccinated with a high-dose poly-epitope vaccine. Cancer immunology, immunotherapy : CII. 2010;59(6):863-73.

23. O'Hara GA, Duncan CJ, Ewer KJ, Collins KA, Elias SC, Halstead FD, et al. Clinical assessment of a recombinant simian adenovirus ChAd63: a potent new vaccine vector. The Journal of infectious diseases. 2012;205(5):772-81.

24. Lohr V, Rath A, Genzel Y, Jordan I, Sandig V, Reichl U. New avian suspension cell lines provide production of influenza virus and MVA in serum-free media: studies on growth, metabolism and virus propagation. Vaccine. 2009;27(36):4975-82.

25. Jordan I, Vos A, Beilfuss S, Neubert A, Breul S, Sandig V. An avian cell line designed for production of highly attenuated viruses. Vaccine. 2009;27(5):748-56.

26. Lee LY, Ha do LA, Simmons C, de Jong MD, Chau NV, Schumacher R, et al. Memory T cells established by seasonal human influenza A infection cross-react with avian influenza A (H5N1) in healthy individuals. J Clin Invest. 2008;118(10):3478-90.
